# Circovirus Rep evades immune restriction by disrupting cGAS oligomerization and phase separation

**DOI:** 10.1371/journal.ppat.1013244

**Published:** 2025-06-16

**Authors:** Hongyan Yin, Zhenchao Zhao, Ye Yuan, Minjie Li, Lvye Chai, Weiyu Qu, Yan Ya, Haiwei Wang, Xin Li

**Affiliations:** 1 National Key Laboratory of Veterinary Public Health and Safety, College of Veterinary Medicine, China Agricultural University, Beijing, China; 2 Key Laboratory of Animal Epidemiology of the Ministry of Agriculture and Rural Affairs, College of Veterinary Medicine, China Agricultural University, Beijing, China; 3 State Key Laboratory of Animal Disease Control, Harbin Veterinary Research Institute, Chinese Academy of Agricultural Sciences, Harbin, China; Florida State University, UNITED STATES OF AMERICA

## Abstract

Cyclic GMP-AMP synthase (cGAS) is a key sensor of double-stranded DNA (dsDNA), initiating oligomerization and phase separation to drive immune responses against pathogens and endogenous damage. Porcine circovirus (PCV) induces immunosuppression, heightening susceptibility to secondary infections, but the underlying mechanisms remain unclear. Here, we report PCV type 2d (PCV2d) infection fails to induce type I interferons (IFN-I) and significantly suppresses IFN-I production upon poly (dA:dT) stimulation in a dose-dependent manner. Mechanistically, the replication-related protein (Rep) proteins of PCV2, PCV3 and PCV4 inhibit cGAS-mediated IFN-I induction by competitively binding dsDNA, thereby disrupting cGAS oligomerization and phase separation. Interestingly, Rep also suppresses mitochondria DNA-induced cGAS activation. We further identify Rep residues Q12 and R199-W202 as key regions facilitating dsDNA binding. Our findings reveal a previously unrecognized mechanism by which circovirus Rep antagonizes cGAS activation, providing new insights into PCV-induced immunosuppression.

## Author summary

The cGAS-STING pathway is pivotal for antiviral immunity by sensing cytosolic DNA to trigger type I interferon (IFN-I) responses. Porcine circovirus type 2 (PCV2), a major swine pathogen, induces immunosuppression. While prior studies focused on the capsid protein (Cap), the role of replication-related protein (Rep) in immune evasion had been unknown. Here, we demonstrate that Rep proteins of PCV2d, PCV2a, PCV3 and PCV4 suppress IFN-I by competitively binding dsDNA, disrupting cGAS oligomerization and phase separation. Rep also inhibits mitochondrial DNA-induced cGAS activation. The residues Q12 and R199-W202 of PCV2d Rep are critical for facilitating dsDNA binding. This study uncovers a novel immune evasion strategy where circovirus Rep antagonizes cGAS, bypassing host defenses. These findings advance our understanding of PCV-induced immunosuppression and underscore Rep’s antagonism of cGAS activation.

## Introduction

Cyclic GMP-AMP synthase (cGAS) is a key cytosolic DNA sensor that detects cytosolic double-stranded DNA (dsDNA) from pathogens or damaged mitochondria, catalyzing the synthesis of 2’3’-cyclic GMP-AMP (2’,3’-cGAMP) [1–5]. This asymmetric second messenger binds to and activates the stimulator of interferon genes (STING), which subsequently activates TANK binding kinase 1 (TBK1) to phosphorylate and activate interferon regulatory factor 3 (IRF3), leading to the induction of type I interferons (IFN-I) and IFN-stimulated genes (ISGs) to inhibit viral replication [6]. However, DNA viruses have evolved multiple strategies to evade cGAS-mediated IFN-I innate immune responses. For example, Kaposi’s sarcoma-associated herpesvirus (KSHV) ORF52, a gammaherpesvirus-specific tegument protein, suppresses cGAS enzymatic activity and IFN-I production by binding to both DNA and cGAS [7]. Likewise, ORF52-family proteins from alpha-herpesvirus also can restrict cGAS-DNA phase separation or interact with cGAS to mediate immune evasion, such as VP22 and ORF9 [8,9]. Similarly, the nucleocapsid (N) protein of severe acute respiratory syndrome coronavirus 2 (SARS-CoV-2) impairs cGAS-induced IFN-I signaling by restricting DNA recognition of cGAS through DNA-induced liquid-liquid phase separation (LLPS) [10]. In addition, viral proteins directly interact with cGAS to inhibit its activation. We previously reported that seneca valley virus (SVV) 3C protease cleaves both cytosolic and nuclear porcine cGAS (pcGAS), disrupting pcGAS activation and IFN-I induction [11,12]. Similarly, human cytomegalovirus (HCMV) protein UL31 directly interacts with cGAS, preventing its DNA binding and inhibiting its enzymatic activity [13]. Furthermore, DNA viruses have evolved strategies to counteract cGAS-STING pathway through post-translational modifications of cGAS. For instance, African swine fever virus (ASFV) QP383R suppresses IFN-I production by promoting cGAS palmitoylation [14].

Porcine circovirus type 2 (PCV2) is a typical single-stranded (ssDNA) virus that establishes an immunosuppressive microenvironment, increasing susceptibility to secondary infections by viruses, bacteria, and mycoplasma [15–17]. PCV-associated diseases (PCVDs) cause significant economic losses in the swine industry worldwide, leading to respiratory and enteric diseases, reproductive failure, porcine dermatitis and nephropathy syndrome (PDNS) [18,19]. While porcine circovirus type 1 (PCV1) is nonpathogenic, porcine circovirus type 3 (PCV3) and porcine circovirus type 4 (PCV4) have been newly identified in recent years [20–22]. Among the PCV2 open reading frames (ORFs), ORF1 encodes the replication-related protein (Rep), while ORF2 encodes the capsid protein (Cap) [23]. However, most studies have focused on Cap, leaving a critical gap in understanding Rep’s role in innate immune responses [24,25]. For example, PCV2 Cap and its binding protein globular head domain of C1q receptor (gC1qR) trigger phosphatidylinositol-3-kinase (PI3K)/AKT signaling to phosphorylate pcGAS, abolishing its catalytic activity and promoting its ubiquitination and degradation [26]. However, whether Rep influences cGAS activation remains unknown. PCV2, one of the smallest DNA viruses, lacks an intrinsic replication enzyme system and relies on host DNA replication machinery. It replicates in the nucleus via Rep/Rep’-mediated rolling-circle replication, binding to double- and single-stranded DNA [27,28]. To date, no studies have reported whether PCV Rep inhibits cGAS activation.

In this study, we found that PCV type 2d (PCV2d) infection suppressed IFN-I induction. The Rep proteins of PCV2, PCV3 and PCV4 significantly disrupted cGAS oligomerization and phase separation by competing with DNA binding at both cellular and recombinant protein levels. Mechanistically, PCV2d Rep directly interacted with dsDNA via its Q12 and R199-W202 regions, competing with cGAS-DNA binding in a dose-dependent manner. Furthermore, Rep from PCV2a and PCV4 exhibited similar inhibitory effects on cGAS activation. These findings provide new insights into how PCV2 infection suppresses IFN-I induction and contributes to immunosuppression.

## Results

### PCV2d infection inhibits dsDNA-mediated IFN-β induction

**Fig 1:**
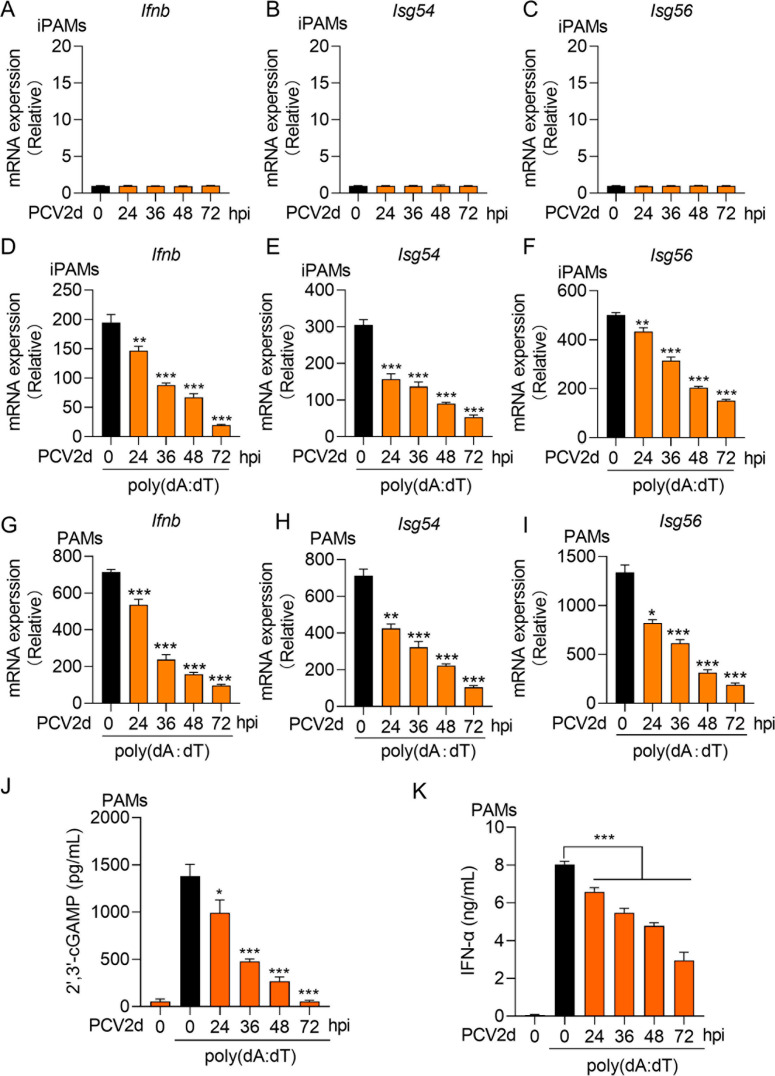
PCV2d infection suppresses dsDNA-mediated IFN- β induction. (A, B and C) RT-PCR analysis of *Ifnb* (A) and downstream *Isg54* (B) and *Isg56* (C) mRNA expression in iPAMs infected with PCV2d (MOI = 1) for 0, 24, 36. 48 and 72 h. (D, E and F) iPAMs were infected with PCV2d (MOI = 1) for indicated time points and then were transfected with poly (dA:dT) at a concentration of 1 µg/ml. After 12 h, cells were harvested for RNA extraction and RT-PCR analysis of *Ifnb* (D) and downstream *Isg54* (E) and *Isg56* (F) mRNA expression. (G, H and I) PAMs were infected with PCV2d (MOI = 1) for indicated time points and then were transfected with poly (dA:dT) (1 µg/ml) for 12 h. Cells were harvested for for RNA extraction and RT-PCR analysis of *Ifnb* (G), *Isg54* (H) and *Isg56* (I) mRNA expression. (J and K) ELISA analysis of the expression of 2’3’-cGAMP (J) and IFN-α (K) in PAMs treated as above. Data are represented as means ± SD from three biological replicates. *p < 0.05, **p < 0.01, ***p < 0.001, Student’s t-test.

PCV type 2d (PCV2d) is known to cause persistent infection and immunosuppression in pigs [25]. Consistent with previous findings, we found that PCV2d infection failed to induce IFN-β and ISGs. Immortalized porcine alveolar macrophages (iPAMs) were infected with PCV2d for 0, 24, 36, 48 and 72 hours, and qPCR analysis showed that there was no transcription of *Ifnb*, *Isg54* and *Isg56*, indicating that PCV2d inactivates IFN-I signaling (Fig 1A-1C). To further investigate its effect on dsDNA-mediated IFN-I induction, iPAMs were infected with PCV2d for 0, 24, 36, 48 and 72 hours, followed by stimulation with poly (dA:dT). The qPCR analysis revealed a significant, time-dependent reduction in the transcription levels of *Ifnb*, *Isg54* and *Isg56* (Fig 1D-1F), suggesting that PCV2d strongly suppresses IFN-I induction upon DNA stimulation. To validate these findings in a physiological context, we isolated primary porcine alveolar macrophages (PAMs) from alveolar lavage fluid and infected them with PCV2d for 0, 24, 36, 48 and 72 hours, followed by poly (dA:dT) treatment for 12 hours. Consistently, PAMs exhibited a time-dependent reduction in the transcription levels of *Ifnb*, *Isg54* and *Isg56* (Fig 1G-1I). Moreover, PCV2d infection significantly decreased the production of 2’,3’-cGAMP and IFN-α secretion, further confirming the suppression of the cGAS-STING pathway (Fig 1J and 1K). Collectively, these results show that PCV2d infection significantly suppresses the expression of IFN-β and ISGs upon poly (dA:dT) stimulation.

### PCV2d Rep attenuates IFN-I production

**Fig 2:**
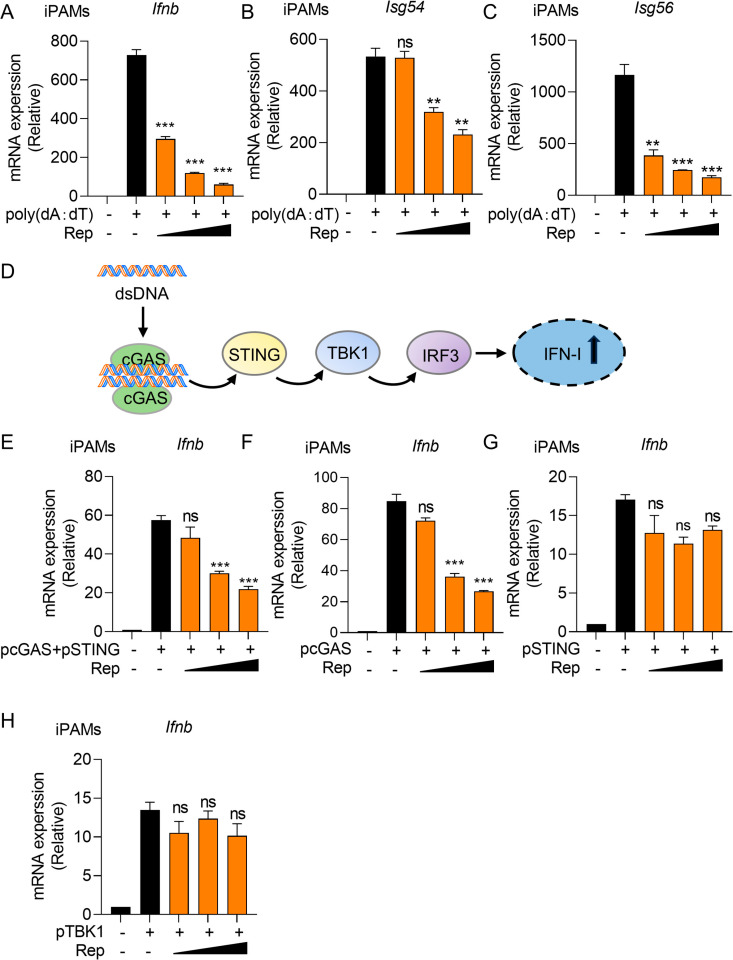
PCV2d Rep inhibits IFN-I production. (A-C) iPAMs were transfected with increasing doses of PCV2d Rep for 24 h. Cells were stimulated with poly (dA:dT) at a concentration of 1 ug/mL. After another 12 h, cells were harvested for RNA extraction and RT-PCR detection of *Ifnb* (A), *Isg54* (B) and *Isg56* (C). (D) Schematic representation of cGAS-STING signaling pathway. (E, F, G, and H) iPAMs were co-transfected pcGAS and pSTING (E), pcGAS (F), pSTING (G) and pTBK1 (H) plasmids and increasing doses of PCV2d Rep plasmid for 24 h. The cells were harvested for RNA extraction and RT-PCR analysis of *Ifnb* mRNA expression. Data are represented as means ± SD from three biological replicates. ns, no significance, **p < 0.01, ***p < 0.001, Student’s t-test.

The replication-related protein (Rep) and the capsid protein (Cap) are the two main structural proteins of PCV. Previous studies have shown that PCV2 Cap inhibits IFN-I induction by targeting cGAS, thereby promoting secondary infections [25]. However, whether PCV Rep plays a role in the IFN-I pathway remains poorly understood. To investigate the effect of Rep on IFN-β expression, iPAMs were transfected with increasing doses of Rep and then stimulated with poly (dA:dT). The results showed that the mRNA levels of *Ifnb*, *Isg54* and *Isg56* were significantly reduced compared to the positive control group (Fig 2A-2C). Similarly, PCV2d Rep remarkably suppressed IFN-β activation in a dose-dependent manner upon poly (dA:dT) and ISD stimulation, suggesting that Rep acts as an antagonist of IFN-I signaling (S1A and 1B Fig).

Upon DNA stimulation, cGAS dimerizes and initiates signal transduction through the synthesis of the second-messenger molecule 2’3’-cGAMP, which binds to and activates STING. Activated STING recruits and activates TBK1, which in turn phosphorylates IRF3 to induce IFN-I and ISGs (Fig 2D). To determine the target of Rep within the cGAS-STING pathway, we examined its effect on IFN-β activation. The results showed the mRNA level of *Ifnb* was reduced upon co-transfection with both porcine cGAS (pcGAS) and porcine STING (pSTING) or pcGAS, but not pSTING or porcine TBK1 (pTBK1) (Fig 2E-2H). Furthermore, Rep did not impair the mRNA level of *Ifnb* in porcine Kidney-15 (PK-15) cells upon 2’,3’-cGAMP stimulation, indicating that Rep does not affect STING function (S1C Fig). Taken together, these results suggest that Rep attenuates IFN-I production at cGAS level.

### Localization of Rep upon PCV2d infection

**Fig 3:**
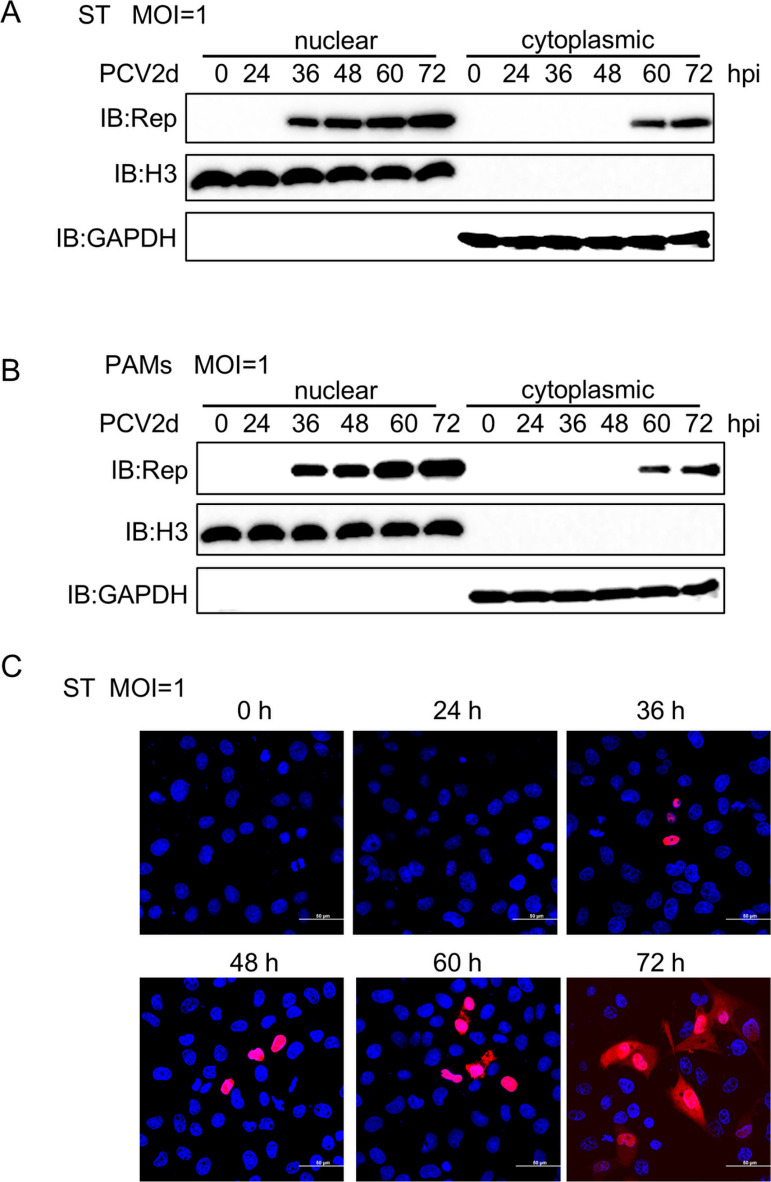
Localization of Rep during PCV2d infection. (A and B) Immunoblotting analysis of PCV2d Rep protein expression in ST cells (A) and PAMs (B) infected with PCV2d (MOI = 1) for 0, 24, 36. 48, 60 and 72 h. (C) Confocal microscopy analysis of PCV2d Rep localization in ST cells infected with PCV2d (MOI = 1) for indicated time points, followed by labeling with Rep-specific primary antibodies and secondary antibodies (red). Cell nuclei were stained with DAPI (blue). The fluorescent signals were observed with confocal immunofluorescence microscopy. Scale bar, 50 μm.

Since cGAS functions as a cytosolic DNA sensor upon viral infection, we next investigated whether Rep protein also localizes in the cytoplasm. To assess Rep localization, we performed nuclear and cytoplasmic fractionation in swine testis (ST) cells and PAMs at different time points after PCV2d infection. The results showed that Rep accumulated in the cytoplasm of ST cells at 60 hours post-infection (hpi) (Fig 3A). Similarly, in PAMs, Rep was primarily localized in the nucleus but appeared in the cytoplasm at 60 hpi (Fig 3B). To further confirm the subcellular localization of endogenous PCV2d Rep, we used a monoclonal antibody (mAb) to detect its distribution in ST cells following PCV2d infection. Consistent with the nuclear and cytoplasmic fractionation results, confocal microscopy showed that Rep was predominantly localized in the nucleus from 36 to 48 hpi, but at 60 hpi, it appeared in the cytoplasm, with its expression increasing further at 72 hpi (Fig 3C). Taken together, these results indicate PCV2d Rep translocates to the cytoplasm upon infection, with detectable cytoplasmic localization at 60 and 72 hpi.

### PCV2d Rep inhibits cGAS oligomerization and phase separation

**Fig 4:**
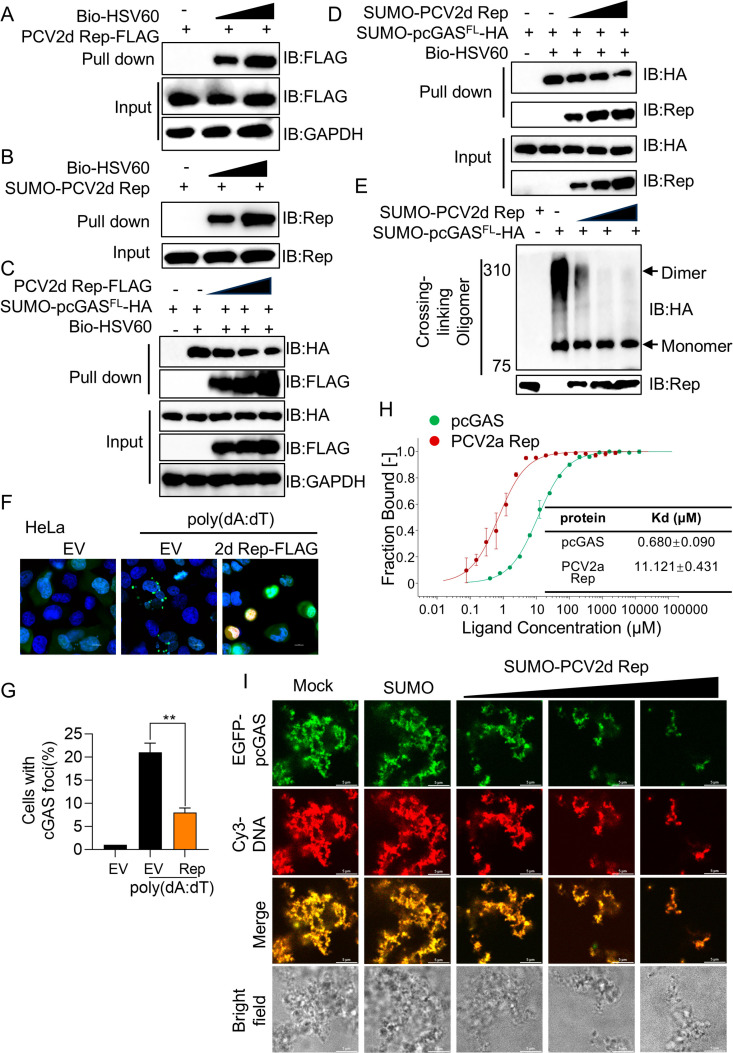
PCV2d Rep disrupts cGAS oligomerization and phase separation. (A) HEK-293T cells were transfected with increasing doses of FLAG- PCV2d Rep for 24 h. The whole cell lysate for streptavidin pull-down was incubated with biotin-HSV60 (40 ug/mL) and pull-down assay of the binding between PCV2d Rep and dsDNA. (B) Pull-down assay for assessment of the interactions between SUMO-PCV2d Rep protein and biotin-HSV60 (40 ug/mL) DNA. After mixing the purified recombinant proteins in NP-40 buffer at 4°C for 6 h, the mixed buffer was immunoprecipitated with Streptavidin mAb. The immunoprecipitated complex was analyzed by immunoblotting with the indicated antibodies. (C) Pull-down assay for assessment of the interactions between the whole cell lysate in HEK-293T cells transfected with increasing doses of FLAG-tagged PCV2d Rep or pcGAS full length protein and biotin-HSV60 (40 ug/mL) DNA. (D) Pull-down assay for assessment of the interactions between SUMO-PCV2d Rep or pcGAS full length protein and biotin-HSV60 (40 ug/mL) DNA. (E) Immunoblotting assay for cGAS complexes incubated with cross-linker between recombinant pcGAS full length and increasing doses of PCV2d Rep proteins. (F) The confocal microscopy analysis of cGAS foci in HeLa cells co-transfected with encoding pcGAS-EGFP and FLAG-PCV2d Rep plasmids for 24 h, followed by stimulation with poly (dA:dT) at a concentration of 1 ug/mL. After another 12 h, cells followed were labelled with FLAG specific primary antibodies and secondary antibodies (red). Cell nuclei were stained with DAPI (blue). The fluorescent signals were observed with confocal immunofluorescence microscopy. Scale bar, 10 μm. (G) The percentage of cells with cGAS foci was quantified. (H) Binding affinities of pcGAS and PCV2a Rep with HSV60 measured by MST. (I) Confocal microscopic analysis of EGFP-pcGAS phase separation, where recombinant EGFP-pcGAS full length (10 μM) protein was incubated with PCV2d Rep protein (10 μM) along with Cy3-45 bp DNA (5 μM). The fluorescent signals were observed with confocal immunofluorescence microscopy. Scale bar, 10 μm. Data are represented as means ± SD from three biological replicates. ns, no significance, **p < 0.01, Student’s t-test.

Previous studies have shown that PCV2 Rep binds to dsDNA [27,29]. To confirm this, we examined if PCV2d Rep binds to biotinylated-HSV60 dsDNA. Co-immunoprecipitation (Co-IP) assay showed that Rep interacted with biotinylated-HSV60 dsDNA (Fig 4A). To further validate this interaction, we purified PCV2d Rep recombinant protein by refolding (S2A and S2B Fig). Consistently, biotinylated-HSV60 dsDNA robustly co-precipitated with the Rep recombinant protein, confirming a direct interaction between Rep and dsDNA (Fig 4B). To investigate whether Rep affects the DNA-binding function of pcGAS, we performed a competition pull-down assay. The results showed that the presence of Rep significantly reduced the interaction between pcGAS and dsDNA in both cellular and recombinant protein levels (Fig 4C and 4D). We employed two complementary assays to assess Rep-mediated restriction of pcGAS oligomerization. First, based on our previous findings, cGAS oligomerization with dsDNA produces a smeared shift band in native agarose gel by electrophoretic mobility shift assay (EMSA) [1]. PCV2d Rep disrupted pcGAS-DNA complex formation in a dose-dependent manner (Fig 4E). The cGAS undergoes oligomerization in the cytosol upon dsDNA stimulation, forming punctate foci that are essential for its activation [30]. PCV2d Rep significantly disrupted the pcGAS foci, indicating inhibition of pcGAS activation (Fig 4F and 4G). To quantify the binding affinity of PCV2a Rep protein for HSV60 dsDNA, we conducted Microscale Thermophoresis (MST) assays using Cy5-labeled HSV60 as the target, testing its interactions with pcGAS and PCV2a Rep, respectively. Due to the suboptimal purity of PCV2d Rep protein purified via inclusion body refolding and the high sequence homology (99%) between PCV2a and PCV2d, we purified soluble-expressed, high-purity Rep protein from PCV2a instead of PCV2d for MicroScale Thermophoresis (MST) experiments (S2C Fig). The results revealed a binding affinity of 11.12 μM for PCV2a Rep with HSV60, compared to 0.68 μM for pcGAS with HSV60 (Fig 4H). Viral proteins may induce phase separation upon binding to dsDNA, potentially disrupting cGAS function, as observed in proteins such as ORF52 and its family members or ZCCHC3 [9,31]. To test whether PCV2a Rep undergoes phase separation with dsDNA, we purified recombinant EGFP-PCV2a Rep protein (S3A Fig). Using the purified EGFP-PCV2a Rep protein, we performed phase separation assays of PCV2a Rep with 45-bp dsDNA at varying salt concentrations (50, 100, 150, 200, 250, and 300 mM NaCl). PCV2a Rep formed a mixture of liquid droplets and gel-like condensate at 150 mM NaCl (S3B Fig). Since fluorescence recovery by photobleaching (FRAP) is a key criterion for characterizing phase separation, we analyzed the gel-like aggregates formed at 150 mM NaCl. FRAP results revealed no fluorescence recovery of PCV2a Rep with DNA after photobleaching, indicating that Rep does not undergo phase separation with dsDNA and instead forms stable complexes with dsDNA (S3C Fig).

Following the methodology of Li et al., who purified human or murine cGAS (hcGAS/mcGAS) recombinant proteins fused with an AcGFP tag for phase separation assays [32], we purified recombinant proteins porcine cGAS (pcGAS) and human cGAS (hcGAS) with an N-terminal EGFP tag (S4 Fig). Using these EGFP-hcGAS and EGFP-pcGAS recombinant proteins, we performed phase separation assays with 45-bp dsDNA at varying salt concentrations (50, 100, 150, 200, 250, and 300 mM NaCl). Human cGAS formed liquid droplets at 150 mM NaCl, whereas pcGAS consistently formed gel-like condensate under the same conditions (S5A Fig), consistent with prior reports [33]. These findings underscore species-specific differences in cGAS phase separation. To further characterize condensate dynamics, we performed fluorescence recovery after photobleaching (FRAP) on pcGAS gel-like condensates at 150 mM NaCl. FRAP analysis revealed rapid fluorescence recovery for both cGAS and DNA after photobleaching, indicating molecular mobility within the condensates of pcGAS and dsDNA (S5B and S5C Fig). Despite their gel-like morphology, pcGAS condensates exhibit dynamic fluidity similar to full-length hcGAS, supporting the conclusion that full-length pcGAS retains DNA-dependent phase separation activity. DNA binding induces liquid-liquid phase separation (LLPS) of cGAS, which is critical for its activation [34,35]. In the presence of Cyanine 3 (Cy3)-labelled 45 bp DNA, EGFP-pcGAS rapidly formed LLPS with enhanced diffusion (Fig 4I, first row). However, the addition of PCV2d Rep caused pcGAS to dissociate from dsDNA, forming small, gel-like droplets with reduced fluorescence recovery, indicating impaired LLPS. PCV2d Rep inhibited pcGAS-dsDNA granule formation in a dose-dependent manner (Fig 4I, last row). As a control, SUMO protein did not affect pcGAS-dsDNA liquid droplets formation (Fig 4I, middle row). Although PCV2a Rep-DNA condensates are static, PCV2a Rep likely suppresses cGAS phase separation through a similar DNA-binding mechanism. Collectively, these findings demonstrate that PCV2d Rep inhibits cGAS activation by suppressing its oligomerization and phase separation.

Rep proteins from PCV2a, PCV3 and PCV4 disrupt cGAS oligomerization and phase separation

**Fig 5:**
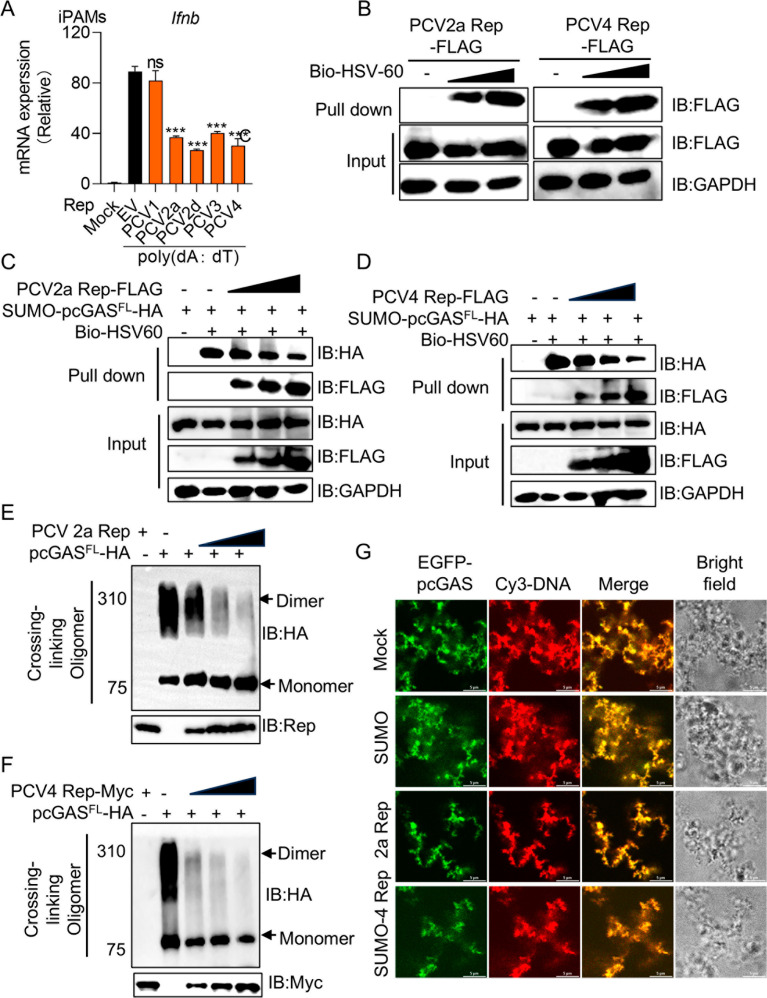
Rep proteins from PCV2a and PCV4 disrupt cGAS oligomerization and phase separation. (A) iPAMs were transfected with Rep of PCV1, PCV2a, PCV2d, PCV3 and PCV4 for 24 h. Cells were stimulated with poly (dA:dT) at a concentration of 1 ug/mL. After another 12 h, cells were harvested for RNA extraction and RT-PCR detection of *Ifnb*. (B) HEK-293T cells were transfected with increasing doses of FLAG-tagged Rep of PCV2a (left) and PCV4 (right) for 24 h. The whole cell lysate for streptavidin pull-down was incubated with biotin-HSV60 (40 ug/mL) and pull-down assay of the binding between Rep and dsDNA. (C and D) Pull-down assay for assessment of the interactions between the whole cell lysate in HEK-293T cells transfected with increasing doses of FLAG-tagged PCV2a (C) and PCV4 (D) Rep or pcGAS full length protein and biotin-HSV60 (40 ug/mL) DNA. (E and F) Immunoblotting assay for pcGAS complexes incubated with cross-linker between recombinant full-length pcGAS and increasing doses of Rep proteins of PCV2a (E) or PCV4 (F). (G) Confocal microscopic analysis of GFP-pcGAS phase separation, where recombinant full-length EGFP-pcGAS (10 μM) was incubated with PCV2a Rep (10 μM) or PCV4 Rep (10 μM) or SUMO (10 μM) proteins, along with Cy3-45 bp DNA (5 μM). The fluorescent signals were observed with confocal immunofluorescence microscopy. Scale bar, 10 μm. Data are represented as means ± SD from three biological replicates. ns, no significance, ***p < 0.001, Student’s t-test.

PCV2 Rep consists of three structural domains: the endonuclease domain (1–118 aa), oligomerization domain (OD, 119–157 aa), and ATPase domain (AD, 158–314 aa). Based on this classification, we constructed a domain organization diagram and performed sequence alignment of Rep proteins from PCV1, PCV2a, PCV2d, PCV3, and PCV4 (S6A Fig). The alignment analysis revealed high sequence conservation between PCV2a and PCV2d Rep proteins, with PCV1 Rep sharing 86% sequence identity with PCV2d Rep. In contrast, PCV3 and PCV4 Rep proteins showed significantly lower homology, with sequence identities of only 46% and 48%, respectively, compared to PCV2d (S6B Fig). To determine whether Rep proteins from other PCV types also inhibit cGAS activation, we synthesized Rep genes from PCV1, PCV2a, PCV3 and PCV4. Similarly, PCV2a, PCV3 and PCV4 Rep significantly reduced the mRNA level of *Ifnb* in iPAMs upon poly (dA:dT) stimulation, suggesting a broad inhibitory role of Rep in cGAS-mediated IFN-β induction (Fig 5A). However, PCV1 Rep had no effect on transcription of *Ifnb*, which may explain why PCV1 is generally considered non-pathogenic. To assess whether the Rep proteins of PCV2a and PCV4 also directly bind dsDNA, HEK-293T cells were transfected with the PCV2a and PCV4 Rep-expressing plasmids, followed by co-precipitation with biotinylated-HSV60 dsDNA. Co-IP analysis showed that the Rep proteins of PCV2a and PCV4 also bound to dsDNA (Fig 5B). To further validate these findings, we purified PCV3 and PCV4 Rep recombinant proteins in *E.*coli (*E.*coli) (S7B and S7C Fig). Consistently, purified Rep recombinant proteins of PCV2a, PCV3 and PCV4 directly bound to HSV60 dsDNA, confirming the results observed in cell-based assays (S8A-S8C Fig).

Since PCV2a, PCV3 and PCV4 Rep directly interact with dsDNA, we next investigated whether they interfere with pcGAS binding to dsDNA using a competition pull-down assay. We observed that PCV2a and PCV4 Rep competed with pcGAS for dsDNA binding, thereby reducing pcGAS-DNA interactions (Fig 5C and 5D). Consistently, in recombinant protein assay, additional Rep proteins of PCV2a, PCV3 and PCV4 inhibited pcGAS binding to dsDNA (S8D-S8F Fig). Since cGAS oligomerization and phase separation are critical for its activation [1], we examined whether Rep proteins of PCV2a and PCV4 affect these processes. We found that the Rep proteins of PCV2a and PCV4 reduced pcGAS oligomerization in a dose-dependent manner (Fig 5E and 5F). To assess phase separation, we incubated recombinant GFP-pcGAS protein (green) with Cy3-DNA (red) and observed robust phase separation of pcGAS and dsDNA (yellow). However, the addition of PCV2a and PCV4 Rep significantly dampened pcGAS condensation, while SUMO protein, used as a control, had no effect, indicating that Rep specifically inhibited the phase separation of cGAS (Fig 5G). Collectively, these results demonstrate that PCV2a and PCV4 Rep also disrupt cGAS oligomerization and phase separation.

### PCV Rep inhibits cGAS activation by self-DNA

**Fig 6:**
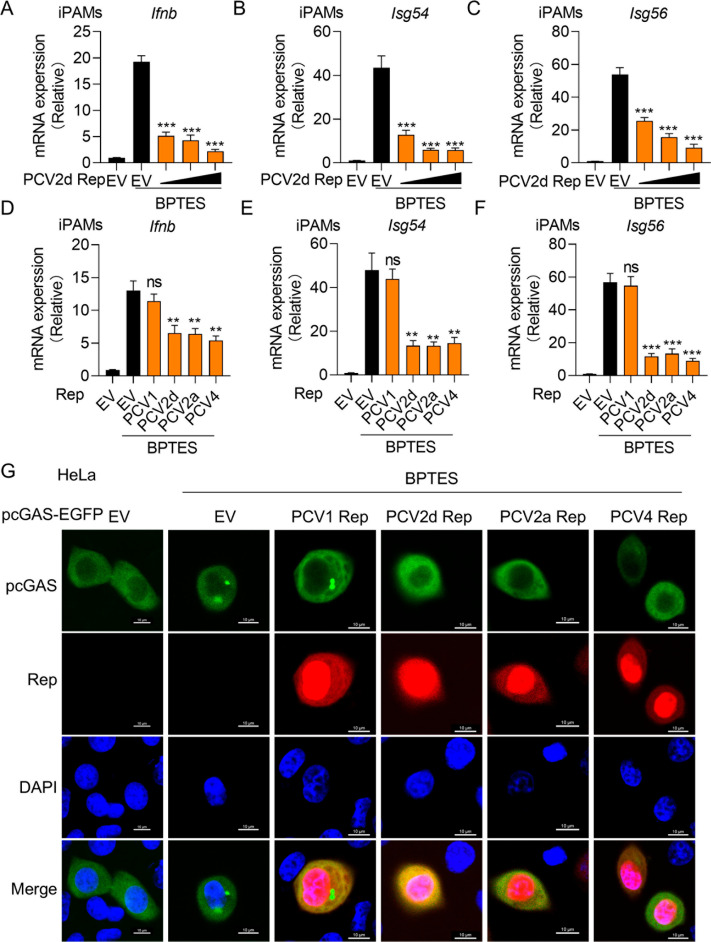
PCV Rep suppresses cGAS activation by self-DNA. (A, B and C) iPAMs were transfected with increasing doses of PCV2d Rep for 24 h. Cells were stimulated with BPTES at a concentration of 10 uM. After another 6 h, cells were harvested for for RNA extraction and RT-PCR detection of *Ifnb* (A), *Isg54* (B) and *Isg56* (C). (D, E and F) iPAMs were transfected with Rep proteins of PCV1, PCV2d, PCV2a and PCV4 for 24 h. Cells were stimulated with BPTES (10 uM) for 6h. Cells were harvested for RNA extraction and RT-PCR detection of *Ifnb* (D), *Isg54* (E) and *Isg56* (F). (G) The confocal microscopy analysis of cGAS foci in HeLa cells co-transfected with encoding pcGAS-EGFP and FLAG-PCV1, PCV2d, PCV2a or PCV4 Rep plasmids for 24 h, were stimulated with BPTES (10 uM). After another 6 h, cells were labelled with FLAG specific primary antibodies and secondary antibodies (red). Cell nuclei were stained with DAPI (blue). The fluorescent signals were observed with confocal immunofluorescence microscopy. Scale bar, 10 μm. Data are represented as means ± SD from three biological replicates. ns, no significance, **p < 0.01, ***p < 0.001, Student’s t-test.

The cGAS can be activated not only by pathogenic DNA but also by self-DNA, such as mitochondrial DNA (mtDNA) or chromosomal DNA [36,37]. Bis-2-(5-phenylacetamido-1,3,4-thiadiazol-2-yl) ethyl sulfide (BPTES) induces mitochondrial dysfunction, leading to mtDNA release into the cytosol, thereby activating cGAS [32]. To determine whether PCV Rep blocks cGAS activation by self-DNA, iPAMs were pre-treated with varying concentrations of BPTES for 6 hours. The mRNA expression of *Ifnb* was highest at 10 μM BPTES treatment, confirming cGAS activation by self-DNA (S9 Fig). However, qPCR analysis revealed that PCV2d Rep significantly reduced the mRNA levels of *Ifnb*, *Isg54 and Isg56* in iPAMs upon BPTES treatment (Fig 6A-6C). Similarly, PCV2a and PCV4 Rep exhibited the same inhibitory effect, whereas PCV1 Rep had no impact (Fig 6D-6F). To further examine whether Rep disrupts cGAS oligomerization upon self-DNA activation, HeLa cells were transfected with EGFP-pcGAS and Rep-FLAG plasmids up to 24 h, followed by BPTES stimulation for 6 hours. Fluorescence imaging showed that PCV2a, PCV2d and PCV4 Rep abolished the foci of pcGAS and dsDNA, indicating disruption of cGAS oligomerization (Fig 6G). Taken together, these results demonstrate that PCV2a, PCV2d and PCV4 Rep proteins effectively suppress cGAS activation by self-DNA.

### Identification of the key DNA-binding regions in PCV2d Rep

**Fig 7:**
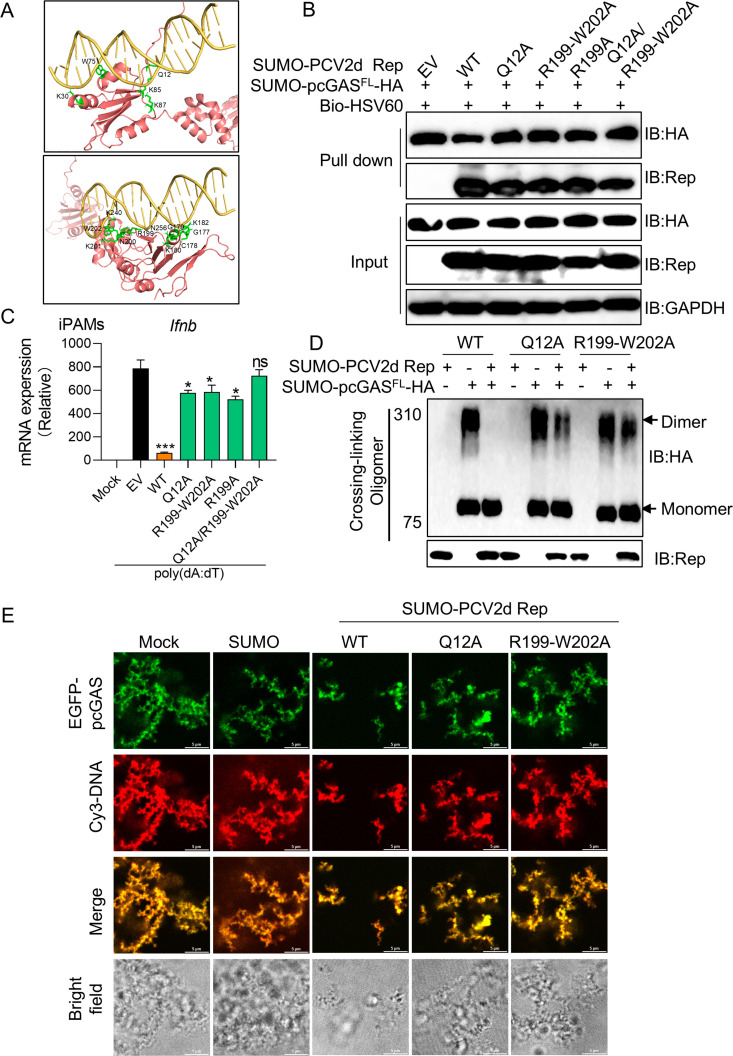
Identification of the key DNA-binding region in PCV2d Rep. (A) Computationally derived binding model of PCV2d Rep with HSV45 dsDNA. (B) Pull-down assay for assessment of the interactions between the whole cell lysate in HEK-293T cells transfected with a plasmid encoding different mutations (wild-type, Q12A, R199-W202A, R199A or Q12A/ R199-W202A) of PCV2d Rep or full-length pcGAS protein and biotin-HSV60 (40 ug/mL) DNA. (C) iPAMs were transfected with a plasmid encoding different mutations (wild-type, Q12A, R199-W202A, R199A or Q12A/ R199-W202A) of PCV2d Rep for 24 h. Cells were stimulated with poly (dA:dT) at a concentration of 1 ug/mL for 12 h. Cells were harvested for RNA extraction and RT-PCR detection of *Ifnb*. (D) Immunoblotting assay for cGAS complexes incubated with cross-linker between recombinant full-length pcGAS and wild-type, Q12A or R199-W202A of PCV2d Rep mutant proteins. (E) Confocal microscopic analysis of EGFP-pcGAS phase separation, where recombinant full-length EGFP-pcGAS (10 μM) was incubated with wild-type, Q12A or R199-W202A of PCV2d Rep mutant proteins or SUMO (10 μM) proteins, along with Cy3-45 bp DNA (5 μM). The fluorescent signals were observed with confocal immunofluorescence microscopy. Scale bar, 10 μm. Data are represented as means ± SD from three biological replicates. ns, no significance, *p < 0.05, ***p < 0.001, Student’s t-test.

To determine the key region of PCV2d Rep responsible for DNA-binding, we predicted the structure of PCV2d Rep (red color) bound to dsDNA (yellow color) HADDOCK2.4 on the website. The model identified a series of positively charged amino acids (green color) likely involved in dsDNA binding, including Q12, K15R16, K87, G177-179, K180, K182, and R199-W202 (Fig 7A). To validate these predictions, we generated PCV2d Rep mutants at these sites and assessed their interaction with dsDNA using pull-down assays. The results showed that only Q12 and R199-W202 were critical for DNA-binding, while mutations at other sites did not affect the interaction (S10A Fig). Additionally, mutations in Q12 and R199-W202 significantly reduced the interaction between pcGAS and dsDNA, suggesting these residues play a key role in cGAS competition for DNA binding (S10B Fig). Given that PCV2d Rep R199-W202 mutant includes R199, N200, K201 and W202, we further examined the effect of single-site mutations within this region. The R199A mutation alone significantly weakened the interaction between PCV2d Rep and dsDNA, as well as the binding between pcGAS and dsDNA (S10C and S10D Fig). Notably, PCV2d Rep Q12 and R199-W202 double mutant significantly impaired its ability to compete with cGAS for dsDNA binding (Fig 7B). Consistently, only the Q12 and R199-W202 double mutant failed to inhibit *Ifnb* mRNA expression, showing levels comparable to the positive control group upon DNA stimulation. In contrast, single Q12, R199-W202, and R199 mutants still exhibited partial inhibition of *Ifnb* transcription, indicating that both Q12 and R199-W202 are required for full inhibition of cGAS-STING mediated IFN-I induction (Fig 7C).

**Fig 8:**
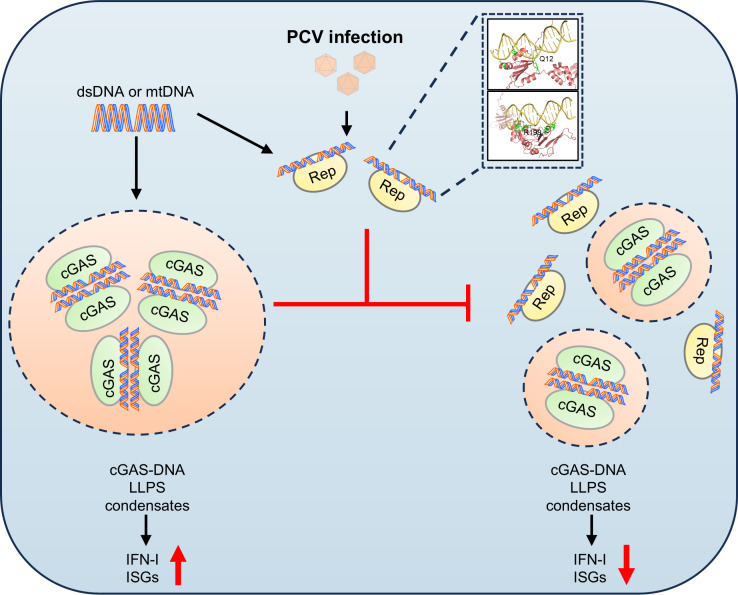
Working model of circovirus Rep-mediated immunosuppression via dsDNA binding and pcGAS inhibition. During pathogenic PCV infection, cGAS binds dsDNA from PCV or released mtDNA, triggering liquid-liquid phase separation (LLPS) condensate formation and subsequent IFN-I production. To establish an immunosuppressive environment, circovirus Rep protein is expressed in the cytoplasm, where it binds dsDNA, blocking recognition and activation, thereby suppressing the antiviral immune responses mediated by IFN-I and ISGs.

To further confirm the role of PCV2d Rep mutations in regulating pcGAS, we expressed and purified Q12 and R199-W202 mutant recombinant proteins (S11A and S11B Fig). However, low expression level of the PCV2d Rep double mutant protein prevented further biochemical assays. Next, we assessed whether PCV2d Rep mutations affect pcGAS oligomerization using a chemical cross-linking assay. Wild-type PCV2d Rep significantly reduced the oligomerization of pcGAS bound to ISD45, but this effect was abolished in the Q12 and R199-W202 mutant groups (Fig 7D). Similarly, we tested whether PCV2d Rep mutations affect DNA‐triggered LLPS of cGAS. Compared to wild-type Rep, fluorescence assays revealed that Q12 and R199-W202 mutations failed to inhibit the formation of cGAS-DNA condensates (Fig 7E). Taken together, these results demonstrate that Q12 and R199-W202 are critical for PCV2d Rep-mediated DNA binding (Fig 8). Loss of these key residues abolishes Rep’s ability to disrupt DNA binding, oligomerization and phase separation of pcGAS, thereby preventing pcGAS-mediated IFN-I suppression (Fig 8).

## Discussion

PCV2 infection induces immunosuppression in piglets, increasing susceptibility to secondary infections [38,39]. Consistent with this, we found PCV2d infection abolished IFN-β production. Upon poly (dA:dT) stimulation, the mRNA levels of *Ifnb*, *Isg54 and Isg56* were significantly reduced in PCV2d-infected cells. Supporting this observation, Huang et al. previously reported PCV2 infection remarkedly promoted the replication of porcine parvovirus and pseudorabies virus [26].

The cGAS-STING pathway is crucial for IFN-I production, which helps inhibit PCV2 infection. However, PCV2 has evolved strategies to counteract cGAS-STING-mediated IFN-I induction. For example, PCV2 Cap inhibits IFN-I signaling by antagonizing the signal transduction of the Janus kinase-signal transducer and activator of transcription (JAK-STAT) pathway during the early phase of infection [40]. Until now, no studies have reported whether PCV Rep directly inhibits the cGAS-STING pathway. In this study, we demonstrate that PCV2d Rep suppresses the mRNA levels of *Ifnb,* Isg54 and *Isg56* upon poly (dA:dT) stimulation in a dose-dependent manner. Mechanistically, PCV2d Rep binds directly to dsDNA and disrupts cGAS oligomerization and phase separation by competing for DNA binding. Importantly, this inhibitory effect is conserved among Rep proteins from PCV2a, PCV2d, PCV3 and PCV4.

PCV is a single-stranded circular DNA that replicates via rolling-circle replication (RCR) [27]. Rep is essential for converting ssDNA into dsDNA, initiating, progressing, and completing RCR of the genome, making it a potential target for virus-specific antiviral drug development. Indeed, we confirmed PCV2d, PCV2a and PCV4 Rep proteins directly bind dsDNA. This dsDNA-binding ability likely explains why PCV2d Rep inhibits cGAS activation by competing for dsDNA binding. Since cGAS is a cytosolic DNA sensor, we examined whether PCV2 Rep also localizes to the cytoplasm during infection. Consistently, PCV2d Rep predominantly accumulates in the cytoplasm at 60 and 72 hours post-infection. Furthermore, PCV2d Rep interacts with dsDNA and competes with cGAS for DNA binding, explaining its ability to suppress cGAS activation. cGAS is shown to trans-locate into the nucleus in cells over-expressing PCV Rep protein, which may represent an additional layer of immune evasion (Fig 4F). In 2021, Zhao et al. demonstrated that nuclear cGAS binds to chromatin via interaction with the H2A-H2B dimer of the nucleosome, immobilizing cGAS and preventing its activation by nearby DNA [41]. In our study, Rep-induced nuclear redistribution of cGAS may complement its direct competition for dsDNA binding, further suppressing cytoplasmic cGAS activity. As an immunosuppressive pathogen, PCV2 has evolved multiple strategies to enhance susceptibility to secondary infections including viruses and bacteria. For example, PCV2 facilitates bacterial infection *in vitro* by reprogramming macrophage, and Cap directly inhibits epigenetic histone modification [16]. Additionally, PCV2/*Streptococcus suis Serotype 2* (SS2) co-infection enhances SS2 intracellular survival while suppressing the inflammatory response by reducing reactive oxygen species (ROS) production in swine tracheal epithelial cells (STEC) [38]. By the time secondary infections occur, Rep has already accumulated in the cytosol (≥60 hpi) and could directly interact with cytosolic dsDNA to suppress cGAS activation. Wu. et.al. found that IFN-α and IFN-β were barely detected in serum of PCV2-infected piglets. Upon PPV infection, the levels of serum IFN-α and IFN-β in PCV2-infected piglets were lower than that in the piglets without PCV2 infection [42]. Additionally, Wang et al. also demonstrated that PCV2 significantly inhibits IFN-β and ISGs production during the secondary infection, such as PPV and PRV [26]. This aligns with our model, where cytosolic Rep plays a critical role in antagonizing cGAS during mtDNA-driven activation. Upon BPTES-induced mtDNA release, Rep proteins from PCV2d, PCV2a and PCV4 impaired the mRNA levels of *Ifnb, Isg54* and *Isg56* in a dose-dependent manner and abrogated cGAS activation by mtDNA. Thus, our data support that PCV2d Rep competes with cGAS for dsDNA binding, contributing to an immunosuppressive environment.

Previous structural studies have identified numerous residues in the N terminus of Rep involved in dsDNA binding, including Gly10, Gln12, His14, Arg16, Asn63, Phe64, Lys66, Lys67, Asn71, Lys72, Val73, Asp90, Arg111, Gln115, and Arg116 [29]. Another study revealed that PCV2 Rep interacts with ssDNA and dsDNA, with Arg199 playing a key role in ssDNA binding [27]. Expanding on these findings, we performed all-atom molecular dynamics (MD) simulations, demonstrating that PCV2d Rep binds dsDNA primarily through both Q12 and R199-W202 regions. However, cGAS apparently binds to DNA more efficiently than PCV2d Rep (Fig 4G), raising the question of whether PCV2d Rep inhibits cGAS activity solely through DNA binding. We investigated the role of DNA binding in cGAS inhibition using a PCV2d Rep Q12/R199A mutant. The mutant’s loss of DNA-binding capacity significantly impaired its ability to inhibit cGAS, confirming that DNA binding by PCV2d Rep is critical for cGAS inhibition (Fig 7B). Similarly to observations with KSHV ORF52, ORF52 exhibits lower DNA-binding affinity than cGAS, yet it can still inhibit cGAS activity by sequestering DNA through direct binding [7]. At 150 mM NaCl, PCV2a Rep formed a mixture of droplets and gel-like condensate. while PCV2a Rep does not undergo phase separation with dsDNA and instead forms stable, non-dynamic complexes with dsDNA, may still suppress cGAS phase separation via this binding mechanism.

In summary, our study reveals that PCV Rep binds dsDNA and disrupts cGAS oligomerization and phase separation, leading to the suppression of cGAS-mediated IFN-I induction. This novel mechanism contributes to PCV2-mediated immunosuppression, facilitating secondary infections and viral persistence.

## Materials and methods

### Ethics statement

Porcine Alveolar Macrophages (PAMs) were isolated following the protocols, which were approved by the Committee on the Ethics of Animal Experiments of the Harbin Veterinary Research Institute (HVRI) of the Chinese Academy of Agricultural Sciences (CAAS) and the Animal Ethics Committee of Heilongjiang Province, China. The license number associated with this research protocol was 231017–01-GR.

### Cells and virus

iPAMs (Immortalized porcine alveolar macrophages), PK-15 cells (Porcine kidney-15), ST cells (Swine testis cells), HEK-293T cells (Human embryonic kidney cells) and HeLa cells were cultured in Dulbecco’s Modified Eagle Medium (DMEM) (MACGENE, #CM10013) containing 10% fetal bovine serum (FBS) (PlantChemMed, #PC-00001) at 37°C with 5% CO_2_, whereas primary porcine alveolar macrophages (PAMs) from 1-mo-old specific pathogen-free (SPF) piglets were maintained in Roswell park memorial institute (RPMI)1640 medium. The porcine circovirus type 2d (PCV2d) virus was stored in our laboratory.

### Antibodies

The following antibodies were used in this study. Rabbit anti-Rep polyclonal antibody (pAb) (#GTX133638) was purchased from GeneTex (Irvine, CA, USA). Mouse anti-Flag monoclonal antibody (mAb) (#F1804) was obtained from Sigma-Aldrich (MO, USA). Rabbit anti-GAPDH pAb (#10494–1-AP), rabbit anti-HA pAb (#51064–2-AP), mouse anti-Myc mAb (#10828–1-AP), coraLite594-conjugated goat anti-mouse IgG(H + L) (#SA00013–3) and coraLite594-conjugated goat anti-rabbit IgG(H + L) (#SA00013–4) were purchased from Proteintech Group (Chicago, IL). Mouse anti IgG HRP-linked antibody and anti-rabbit IgG HRP-linked antibody were purchased from Cell Signaling Technology (Beverly, MA).

### Plasmids

The N-terminal FLAG tagged PCV2d Rep (GenBank: ON500676.1) (wild-type, Q12A, K15A/R16A, K87A, G177-179A, K180A/K182A, R199-W202A, R199A, N200A, K201A or W202A mutations), PCV1 Rep (GenBank: KC447455.1), PCV2a Rep (GenBank: FJ870968.1), PCV3 Rep (GenBank: MN788125.1) and PCV4 Rep (GenBank: MT311854.1) were cloned into pcDNA3.1 vector. The C-terminal Myc tagged PCV2d Rep (wild-type, Q12A or R199-W202A mutations), PCV2a Rep, PCV3 Rep and PCV4 Rep were cloned into His6-SUMO-pET28a vector. The N-terminal EGFP tagged full-length pcGAS and hcGAS were cloned into His6-pET28a vector Mutant plasmids of PCV2d Rep were constructed following the manufacturer’s instructions of Mut Express II Fast Mutagenesis Kit V2 (Vazyme, #C1002). The pcDNA3.1-HA-pcGAS, pcDNA3.1-HA-pSTING and pcDNA3.1-HA-pTBK1 were stored in our laboratory. The recombinant protein full-length pcGAS was stored in our laboratory.

### Immunoblotting

HEK-293T cells were transfected with indicated plasmids using Lipofectamine 2000 (Invitrogen, #11668019) for 24 h post transfection, cells were lysed with IP lysis buffer (Beyotime, #P0013) for 30 min on ice. Whole-cell lysates or immunoprecipitated extracts were then separated by 10% SDS-PAGE gels (EpiZyme, #PG112) and transferred onto PVDF membrane (Millipore) for immunoblotting with specific antibodies.

### Pull-down assay

HEK-293T cells were cultured in 6-well plates and transfected with indicated plasmids. After 24 h of transfection, cells were washed twice with pre-cooled PBS, collected and lysed with IP lysis buffer (Beyotime, #P0013) for 30 min on ice. Cell lysates were harvested by centrifugation at 12,000 rpm for 10 min at 4°C, and incubated with biotinylated nucleic acids (synthesized by Sangon Biotech, Shanghai, China) for 4 h at 4°C. Then the mixture containing protein and biotinylated HSV60 (5’-biotin-TAAGACACGATGCGATAAAATCTGTTTGTAAAATTTATTAAGGGTACAAATT-GCCCTAGC-3’) were incubated with streptavidin magnetic beads (Changzhou Smart-Lifesciences Biotechnology, # SM017010) for another 2 h. After washing five times, the beads were denatured with SDS buffer at 95°C for 10 min and subjected to immunoblotting analysis [43].

### Protein expression and purification

PCV1, PCV2a, PCV3, PCV4 Rep proteins, Q12A and R199-W202A of PCV2d Rep mutant proteins, EGFP-PCV2a Rep, EGFP-pcGAS and EGFP-hcGAS protein were obtained as previously described [11], *E. coli* BL21 (DE3) cells harboring His6-SUMO-pET28a-PCV1 Rep-Myc, His6-SUMO-pET28a-PCV2a Rep-Myc, His6-SUMO-pET28a-PCV2d Rep Q12A-Myc, His6-SUMO-pET28a-PCV2d Rep R199-W202A-Myc, His6-SUMO-pET28a-PCV3 Rep-Myc, His6-SUMO-pET28a-PCV4 Rep-Myc, His6-SUMO-pET28a-EGFP-PCV2a Rep, His6-SUMO-pET28a-EGFP-pcGAS and His6-SUMO-pET28a-EGFP-hcGAS plasmids were grown in LB medium with 50 µg/ml Kanamycin. When optical density at 600 nm (OD600) reached at 1.0, the proteins were induced overnight at 16°C with 0.01 mM isopropyl β-D-1-thiogalactopyranoside (IPTG, #18070). The bacteria were lysed in buffer containing 0.05 M Tris-HCl (pH 8.0), 0.3 M NaCl. The proteins were then purified by Ni-NTA beads followed by washing with the buffer containing 0.02 M Tris-HCl (pH 7.5), 0.5 M NaCl and 0.025 M imidazole and eluting with the buffer containing 0.02 M Tris-HCl (pH 7.5), 0.15 M NaCl and 250mM imidazole. The His6-SUMO tag was removed by SUMO protease overnight at 4°C. Then, the proteins were further purified by Hiload 16/600 Superdex 200pg gel-filtration chromatography with running buffer containing 20 mM Tris-HCl (pH 7.5), 150 mM NaCl and then purified by resource S ion exchange with buffer A containing 10 mM Tris-HCl (pH 8.0), 100 mM NaCl and buffer B containing 10 mM Tris-HCl (pH 8.0), 1 M NaCl. The purified proteins were concentrated to ~12 mg/ml and frozen in liquid nitrogen immediately. All purified proteins were stored in running buffer with 5 mM dithiothreitol (DTT).

Recombinant SUMO-PCV2d Rep protein was refolded by 7 M Guaiacol hydrochloride and dialyzed against multiple changes of phosphate-buffered saline.

### Immunofluorescence and confocal immunofluorescence assay

HeLa and ST cells were seeded into 24-well plates containing slides. When confluence reached up to 60–70%, the cells were transfected with the indicated plasmids, and then were washed with PBS and fixed for 30 min in 4% paraformaldehyde (Solarbio, #P1110). Cells were then permeabilized for 5 min with 0.1% Triton X-100 and then blocked with 5% bovine serum albumin (BSA) (Sigma-Aldrich, #A7906-100G) for 0.5 h. Cells were incubated with the appropriate primary antibodies for 1 h and then stained with Alexa Fluor 594- conjugated secondary antibodies for 1 h. Images were collected and analyzed by NIS-Elements AR.

### Enzyme-linked immunosorbent assay (ELISA)

PAMs were seeded into 6-well plates and were infected with PCV2d virus (MOI = 1) for 0, 24, 36, 48 and 72 h, then followed stimulated with poly (dA:dT) for 12 h. The secreted 2’3’-cGAMP (501700, Cayman) and IFN-α (819120921, Thermo Scientific) in cell culture medium from PCV2d-infected cells were analyzed with ELISA kits [44].

### Chemical cross-linking of recombinant proteins

Recombinant pcGAS protein (5 μg) was incubated with recombinant Rep protein or its mutant variants (5, 10, 15 μg) in the cGAMP synthesis buffer (20 mM HEPES, pH 7.5; 5 mM MgCl_2_; 2 mM ATP and 2 mM GTP) at 25°C for 15 min before dioctyl sodium sulfosuccinate (DSS) (0.5 mM) added and incubated for another 10 min. The reactions were stopped by adding loading buffer (2-mercaptoethanol free) and the samples were analyzed with immunoblotting.

### Liquid-liquid phase separation (LLPS) assay in *vitro*

The purified recombinant EGFP-pcGAS protein was mixed at indicated concentration with LLPS buffer (1 mg/mL BSA, 20 mM Tris-HCl and 150 mM NaCl), followed by incubating with indicated Cy3-dsDNA for 5 min at 37°C. The mixture was pipetted onto glass bottom dish and images were collected and analyzed by NIS-Elements AR.

### Reverse transcription and quantitative real-time PCR (RT-qPCR)

Total RNA was extracted by a simple total RNA kit (TIANGEN, #DP419) and was reversely transcribed to cDNA using HiScript II Q RT SuperMix (Vazyme, #R223-01) according to the manufacturer’s protocol. qPCR was performed in triplicated determinants with 2 × Taq Pro Universal SYBR qPCR Master Mix (Vazyme, #Q712-02) on a LightCycler 480 II system (Roche, Switzerland). Relative gene expression levels were determined based on the cycle threshold (ΔΔCT) method and normalized to glyceraldehyde-3-phosphate dehydrogenase (GAPDH) expression. The sequences of qPCR primer sequences were as follows: sus GAPDH-F: ACATGGCCTCCAAGGAGTAAGA, sus GAPDH-R: GACGCCTGCTTCACCACCTTCT; sus *Ifnb*-F: TGCATCCTCCAAATCGCTCT, sus *Ifnb*-R: ATTGAGGAGTCCCAGGCAAC; sus *Isg54*-F: GCACAGCAATCATGAGTGAGAC, sus *Isg54*-R: CTGGCCCCTGCAGTCTTTTA; sus *Isg56*-F: TCCGACACGCAGTCAAGTTT, sus *Isg56*-R: TGTAGCAAAGCCCTGTCTGG.

### Computational modeling

The all-atom MD simulations of PCV2d Rep (PDB: 7LAS) and 18 bp dsDNA (PDB: 4LEYDNA) were performed using HADDOCK2.4 on the website (https://wenmr.science.uu.nl/haddock2.4/).

### NE-PER nuclear and cytoplasmic extraction

Fractions of cytoplasmic and nuclear extracts, where necessary, were prepared using the NE-PER nuclear and cytoplasmic extraction reagents (78835, Thermo Scientific) following the manufacturer’s instructions. SDS-PAGE (15% gels) and immunoblotting were carried out as described previously.

### Statistical data analysis

All the graphs and relevant statistical tests used in the work were created by GraphPad software (v9.0). Data were expressed as mean ± SD and statistically analyzed with a two-tailed unpaired Student’s t-test. The *p* values of <0.05 were considered significant. *p < 0.05, **p < 0.01, ***p < 0.001. ns, no significance.

## Supporting information

S1 Fig PCV2d Rep suppresses cGAS-mediated IFN-β production. (A to C) PK-15 cells were transfected with increasing doses of PCV2d Rep for 24 h. Cells were stimulated with poly (dA:dT) (1 μg/mL) (A), ISD (2 μg/mL) (B) and 2’3’-cGAMP (1 μg/mL) (C). After another 12 h, cells were harvested for for RNA extraction and RT-PCR analysis of *Ifnb* expression. Data are represented as means ± SD from three biological replicates. ns, no significance, ***p < 0.001, Student’s t-test.
(TIF)

S2 Fig Purification of Rep proteins from PCV2d and PCV2a. (A) Purification of refolding of full-length PCV2d Rep protein in *vitro*. (B) The detection of refolding of SUMO-tagged PCV2d Rep protein by gel-filtration chromatography. (C). Purification of PCV2a Rep protein by gel-filtration chromatography.
(TIF)

S3 Fig Exploring phase separation conditions for PCV2a Rep. (A) Purification of EGFP-tagged PCV2a Rep protein by gel-filtration chromatography. (B) Phase separation of EGFP-PCV2a Rep with Cy3–45 bp DNA. (C) Fluorescence recovery of the PCV2a Rep-DNA condensate after photobleaching (FRAP). Bleaching was performed at the indicated time points after PCV2a Rep (10 μM) and DNA (5 μM) were mixed and the recovery was occured at 25 °C. Scale bar, 5 μm. The maximal fluorescence intensity was normalized to 1.
(TIF)

S4 Fig Purification of EGFP-pcGAS and EGFP-hcGAS proteins. (A and B) Purification of EGFP-tagged full-length pcGAS protein by gel-filtration chromatography (A) and ion exchange (B). (C and D) Purification of EGFP-tagged full-length hcGAS protein by gel-filtration chromatography (C) and ion exchange (D).
(TIF)

S5 Fig Exploring phase separation conditions for pcGAS. (A) Phase separation was induced with recombinant hcGAS (10 μM) and pcGAS (10 μM) and 45 bp dsDNA (5 μM) in buffer with varying salt concentration. Scale bar, 10 μm. (B and C) Fluorescence recovery after photobleaching (FRAP) of pcGAS–DNA phase-separated condensates. Bleaching was performed at the indicated time points after pcGAS (10 μM) and DNA (5 μM) were mixed and the recovery was allowed to occur at 25 °C. Scale bar, 5 μm. The maximal fluorescence intensity was normalized to 1. The statistical values are shown in C.
(TIF)

S6 Fig Amino acid sequence alignment of PCV Rep proteins. (A and B) PCV1 Rep (Genbank: KC447455.1), PCV2a Rep (Genbank: FJ870968.1), PCV2d Rep (Genbank: ON500676.1), PCV3 Rep (Genbank: MN788125.1) and PCV4 Rep (Genbank: MT311854.1) were aligned by Clustal Omega algorithm. Schematic of Rep with the endonuclease domain (pastel yellow), oligomerization domain (pastel red), and ATPase domain (pastel blue) labeled. (B). Comparison of amino acids identity (%) among different PCV Rep proteins.
(TIF)

S7 Fig Purification of Rep proteins from PCV3 and PCV4. (A) The table of molecular weights of PCV2a, SUMO-PCV3 Rep, and SUMO-PCV4 Rep recombinant proteins using NovoPro (https://novopro.cn/tools/protein-sds-page-mw.html). (B and C) Purification of SUMO-PCV3 Rep (B) and SUMO-PCV4 Rep (C) protein by gel-filtration chromatography.
(TIF)

S8 Fig PCV2a, PCV3 and PCV4 recombinant Rep proteins bind dsDNA to inhibit cGAS activity. (A-C) Pull-down assay for assessment of the interactions between PCV2a (A), PCV3 (B) and PCV4 (C) Rep protein and biotin-HSV60 (40 μg/mL) DNA. After mixing the purified recombinant proteins in NP-40 buffer at 4°C for 6 h, the mixed buffer was immunoprecipitated with Streptavidin mAb. The immunoprecipitated complex was analyzed by immunoblotting with the indicated antibodies. (D-F) Pull-down assay for assessment of the interactions between PCV2a Rep (C), SUMO-PCV3 Rep-Myc (E) or SUMO-PCV4 Rep-Myc (F) or pcGAS full length protein and biotin-HSV60 (40 μg/mL) DNA.
(TIF)

S9 Fig The mRNA expression of *Ifnb* in iPAMs following BPTES treatment. iPAMs were stimulated with BPTES at 5, 10 or 20 μM for 6 h, then were harvested for RNA extraction and RT-PCR analysis of *Ifnb* expression. (TIF)

S10 Fig PCV2d Rep binds dsDNA via Q12 and R199 sites. (A) Pull-down assay for assessment of the interactions between the whole cell lysate in HEK-293T cells transfected with a plasmid encoding different mutations (wild-type, Q12A, K15A/R16A, K87A, G177-179A, K180A/K182A or R199-W202A) of PCV2d Rep and biotin-HSV60 (40 μg/mL) DNA. (B) Pull-down assay for assessment of the interactions between the whole cell lysate in HEK-293T cells transfected with a plasmid encoding different mutations (wild-type, Q12A, K15A/R16A, K87A, G177-179A, K180A/K182A or R199-W202A) of PCV2d Rep or full-length pcGAS protein and biotin-HSV60 (40 μg/mL) DNA. (C) Pull-down assay for assessment of the interactions between the whole cell lysate in HEK-293T cells transfected with a plasmid encoding different mutations (wild-type, R199A, N200A, K201A or W202A) of PCV2d Rep and biotin-HSV60 (40 μg/mL) DNA. (D) Pull-down assay for assessment of the interactions between the whole cell lysate in HEK-293T cells transfected with a plasmid encoding different mutations (wild-type, R199A, N200A, K201A or W202A) of PCV2d Rep or full-length pcGAS protein and biotin-HSV60 (40 μg/mL) DNA.
(TIF)

S11 Fig Purification of Q12A and R199-W202A mutant PCV2d Rep proteins. (A and B) Purification of recombinant Q12A (A) and R199-W202A (B) mutation PCV2d Rep proteins by Ni-NTA.
(TIF)

## References

[1] Li X, Shu C, Yi G, Chaton CT, Shelton CL, Diao J, et al. Cyclic GMP-AMP synthase is activated by double-stranded DNA-induced oligomerization. Immunity. 2013;39(6):1019–31. doi: 10.1016/j.immuni.2013.10.019 24332030 PMC3886715

[2] Sun L, Wu J, Du F, Chen X, Chen ZJ. Cyclic GMP-AMP synthase is a cytosolic DNA sensor that activates the type I interferon pathway. Science. 2013;339(6121):786–91. doi: 10.1126/science.1232458 23258413 PMC3863629

[3] Wu J, Sun L, Chen X, Du F, Shi H, Chen C, et al. Cyclic GMP-AMP is an endogenous second messenger in innate immune signaling by cytosolic DNA. Science. 2013;339(6121):826–30. doi: 10.1126/science.1229963 23258412 PMC3855410

[4] Gao P, Ascano M, Wu Y, Barchet W, Gaffney BL, Zillinger T, et al. Cyclic [G(2’,5’)pA(3’,5’)p] is the metazoan second messenger produced by DNA-activated cyclic GMP-AMP synthase. Cell. 2013;153(5):1094–107. doi: 10.1016/j.cell.2013.04.046 23647843 PMC4382009

[5] Ablasser A, Goldeck M, Cavlar T, Deimling T, Witte G, Röhl I, et al. cGAS produces a 2’-5’-linked cyclic dinucleotide second messenger that activates STING. Nature. 2013;498(7454):380–4. doi: 10.1038/nature12306 23722158 PMC4143541

[6] Chen C, Xu P. Cellular functions of cGAS-STING signaling. Trends Cell Biol. 2023;33(8):630–48. doi: 10.1016/j.tcb.2022.11.001 36437149

[7] Wu J, Li W, Shao Y, Avey D, Fu B, Gillen J, et al. Inhibition of cGAS DNA Sensing by a Herpesvirus Virion Protein. Cell Host Microbe. 2015;18(3):333–44. doi: 10.1016/j.chom.2015.07.015 26320998 PMC4567405

[8] Bhowmik D, Du M, Tian Y, Ma S, Wu J, Chen Z, et al. Cooperative DNA binding mediated by KicGAS/ORF52 oligomerization allows inhibition of DNA-induced phase separation and activation of cGAS. Nucleic Acids Res. 2021;49(16):9389–403. doi: 10.1093/nar/gkab689 34387695 PMC8450086

[9] Xu G, Liu C, Zhou S, Li Q, Feng Y, Sun P, et al. Viral tegument proteins restrict cGAS-DNA phase separation to mediate immune evasion. Mol Cell. 2021;81(13):2823-2837.e9. doi: 10.1016/j.molcel.2021.05.002 34015248

[10] Cai S, Zhang C, Zhuang Z, Zhang S, Ma L, Yang S, et al. Phase-separated nucleocapsid protein of SARS-CoV-2 suppresses cGAS-DNA recognition by disrupting cGAS-G3BP1 complex. Signal Transduct Target Ther. 2023;8(1):170. doi: 10.1038/s41392-023-01420-9 37100798 PMC10131525

[11] Yan Y, Wu L, Yuan Y, Wang H, Yin H, Li M, et al. Species-specific cleavage of cGAS by picornavirus protease 3C disrupts mitochondria DNA-mediated immune sensing. PLoS Pathog. 2023;19(9):e1011641. doi: 10.1371/journal.ppat.1011641 37708231 PMC10521975

[12] Wu L, Yan Y, Yuan Y, Zhao Z, Qu W, Huang X, et al. Viral protease binds to nucleosomal DNA and cleaves nuclear cGAS that attenuates type I interferon. mBio. 2025;16(4):e0339524. doi: 10.1128/mbio.03395-24 39998223 PMC11980361

[13] Huang Z-F, Zou H-M, Liao B-W, Zhang H-Y, Yang Y, Fu Y-Z, et al. Human Cytomegalovirus Protein UL31 Inhibits DNA Sensing of cGAS to Mediate Immune Evasion. Cell Host Microbe. 2018;24(1):69-80.e4. doi: 10.1016/j.chom.2018.05.007 29937271

[14] Hao S, Zheng X, Zhu Y, Yao Y, Li S, Xu Y, et al. African swine fever virus QP383R dampens type I interferon production by promoting cGAS palmitoylation. Front Immunol. 2023;14:1186916. doi: 10.3389/fimmu.2023.1186916 37228597 PMC10203406

[15] Ouyang T, Zhang X, Liu X, Ren L. Co-Infection of Swine with Porcine Circovirus Type 2 and Other Swine Viruses. Viruses. 2019;11(2):185. doi: 10.3390/v11020185 30795620 PMC6410029

[16] Zhang W, Fu Z, Yin H, Han Q, Fan W, Wang F, et al. Macrophage Polarization Modulated by Porcine Circovirus Type 2 Facilitates Bacterial Coinfection. Front Immunol. 2021;12:688294. doi: 10.3389/fimmu.2021.688294 34394082 PMC8355693

[17] Niu G, Chen S, Li X, Zhang L, Ren L. Advances in Crosstalk between Porcine Circoviruses and Host. Viruses. 2022;14(7):1419. doi: 10.3390/v14071419 35891399 PMC9315664

[18] Zhang J, Wang P, Xie C, Ha Z, Shi N, Zhang H, et al. Synergistic Pathogenicity by Coinfection and Sequential Infection with NADC30-like PRRSV and PCV2 in Post-Weaned Pigs. Viruses. 2022;14(2):193. doi: 10.3390/v14020193 35215787 PMC8877551

[19] Ge X, Wang F, Guo X, Yang H. Porcine circovirus type 2 and its associated diseases in China. Virus Res. 2012;164(1–2):100–6. doi: 10.1016/j.virusres.2011.10.005 22023739

[20] Zhang H-H, Hu W-Q, Li J-Y, Liu T-N, Zhou J-Y, Opriessnig T, et al. Novel circovirus species identified in farmed pigs designated as Porcine circovirus 4, Hunan province, China. Transbound Emerg Dis. 2020;67(3):1057–61. doi: 10.1111/tbed.13446 31823481

[21] Chen S, Zhang L, Li X, Niu G, Ren L. Recent Progress on Epidemiology and Pathobiology of Porcine Circovirus 3. Viruses. 2021;13(10):1944. doi: 10.3390/v13101944 34696373 PMC8538958

[22] Opriessnig T, Karuppannan AK, Castro AMMG, Xiao C-T. Porcine circoviruses: current status, knowledge gaps and challenges. Virus Res. 2020;286:198044. doi: 10.1016/j.virusres.2020.198044 32502553

[23] Lv Q-Z, Guo K-K, Zhang Y-M. Current understanding of genomic DNA of porcine circovirus type 2. Virus Genes. 2014;49(1):1–10. doi: 10.1007/s11262-014-1099-z 25011695

[24] Lv Q, Wang T, Liu S, Zhu Y. Porcine circovirus type 2 exploits cap to inhibit PKR activation through interaction with Hsp40. Vet Microbiol. 2021;252:108929. doi: 10.1016/j.vetmic.2020.108929 33254057

[25] Du Q, Zhu L, Zhong J, Wei X, Zhang Q, Shi T, et al. Porcine circovirus type 2 infection promotes the SUMOylation of nucleophosmin-1 to facilitate the viral circular single-stranded DNA replication. PLoS Pathog. 2024;20(2):e1012014. doi: 10.1371/journal.ppat.1012014 38394330 PMC10917307

[26] Wang Z, Chen J, Wu X, Ma D, Zhang X, Li R, et al. PCV2 targets cGAS to inhibit type I interferon induction to promote other DNA virus infection. PLoS Pathog. 2021;17(9):e1009940. doi: 10.1371/journal.ppat.1009940 34543359 PMC8483418

[27] Tarasova E, Dhindwal S, Popp M, Hussain S, Khayat R. Mechanism of DNA Interaction and Translocation by the Replicase of a Circular Rep-Encoding Single-Stranded DNA Virus. mBio. 2021;12(4):e0076321. doi: 10.1128/mBio.00763-21 34311576 PMC8406172

[28] Guan S, Tian A, Jing H, Yuan H, Jia H, Shi Y, et al. Crystal structure of the dimerized of porcine circovirus type II replication-related protein Rep’. Proteins. 2023;91(8):1130–9. doi: 10.1002/prot.26498 37171131

[29] Luo G, Zhu X, Lv Y, Lv B, Fang J, Cao S, et al. Crystal Structure of the Dimerized N Terminus of Porcine Circovirus Type 2 Replicase Protein Reveals a Novel Antiviral Interface. J Virol. 2018;92(18):e00724-18. doi: 10.1128/JVI.00724-18 29976661 PMC6146719

[30] Liu Z-S, Cai H, Xue W, Wang M, Xia T, Li W-J, et al. G3BP1 promotes DNA binding and activation of cGAS. Nat Immunol. 2019;20(1):18–28. doi: 10.1038/s41590-018-0262-4 30510222 PMC8276115

[31] Shi M, Jiang T, Zhang M, Li Q, Liu K, Lin N, et al. Nucleic-acid-induced ZCCHC3 condensation promotes broad innate immune responses. Mol Cell. 2025;85(5):962-975.e7. doi: 10.1016/j.molcel.2025.01.027 39983719

[32] Li H, Liu C, Li R, Zhou L, Ran Y, Yang Q, et al. AARS1 and AARS2 sense L-lactate to regulate cGAS as global lysine lactyltransferases. Nature. 2024;634(8036):1229–37. doi: 10.1038/s41586-024-07992-y 39322678

[33] Zhou W, Mohr L, Maciejowski J, Kranzusch PJ. cGAS phase separation inhibits TREX1-mediated DNA degradation and enhances cytosolic DNA sensing. Mol Cell. 2021;81(4):739-755.e7. doi: 10.1016/j.molcel.2021.01.024 33606975 PMC7899126

[34] Du M, Chen ZJ. DNA-induced liquid phase condensation of cGAS activates innate immune signaling. Science. 2018;361(6403):704–9. doi: 10.1126/science.aat1022 29976794 PMC9417938

[35] Xie W, Lama L, Adura C, Tomita D, Glickman JF, Tuschl T, et al. Human cGAS catalytic domain has an additional DNA-binding interface that enhances enzymatic activity and liquid-phase condensation. Proc Natl Acad Sci U S A. 2019;116(24):11946–55. doi: 10.1073/pnas.1905013116 31142647 PMC6575157

[36] Dvorkin S, Cambier S, Volkman HE, Stetson DB. New frontiers in the cGAS-STING intracellular DNA-sensing pathway. Immunity. 2024;57(4):718–30. doi: 10.1016/j.immuni.2024.02.019 38599167 PMC11013568

[37] Zhao C, Ma Y, Zhang M, Gao X, Liang W, Qin Y, et al. Polyamine metabolism controls B-to-Z DNA transition to orchestrate DNA sensor cGAS activity. Immunity. 2023;56(11):2508-2522.e6. doi: 10.1016/j.immuni.2023.09.012 37848037

[38] Wang Q, Zhou H, Fan H, Wang X. Coinfection with Porcine Circovirus Type 2 (PCV2) and Streptococcus suis Serotype 2 (SS2) Enhances the Survival of SS2 in Swine Tracheal Epithelial Cells by Decreasing Reactive Oxygen Species Production. Infect Immun. 2020;88(11):e00537-20. doi: 10.1128/IAI.00537-20 32868342 PMC7573442

[39] Li J, Lv L, Gao Y, Sun Y, Bai J, Wang X, et al. Tetraspanin CD81 serves as a functional entry factor for porcine circovirus type 2 infection. J Virol. 2024;99(2):e0140824. doi: 10.1128/jvi.01408-24 39745447 PMC11853000

[40] Wang Z, Chen J, Zhang Q-G, Huang K, Ma D, Du Q, et al. Porcine circovirus type 2 infection inhibits the activation of type I interferon signaling via capsid protein and host gC1qR. Vet Microbiol. 2022;266:109354. doi: 10.1016/j.vetmic.2022.109354 35085949

[41] Zhao B, Xu P, Rowlett CM, Jing T, Shinde O, Lei Y, et al. The molecular basis of tight nuclear tethering and inactivation of cGAS. Nature. 2020;587(7835):673–7. doi: 10.1038/s41586-020-2749-z 32911481 PMC7704945

[42] Wu X, Wang Z, Qiao D, Yuan Y, Han C, Yang N, et al. Porcine circovirus type 2 infection attenuates the K63-linked ubiquitination of STING to inhibit IFN-β induction via p38-MAPK pathway. Vet Microbiol. 2021;258:109098. doi: 10.1016/j.vetmic.2021.109098 33984793

[43] Xing J, Zhang A, Du Y, Fang M, Minze LJ, Liu Y-J, et al. Identification of poly(ADP-ribose) polymerase 9 (PARP9) as a noncanonical sensor for RNA virus in dendritic cells. Nat Commun. 2021;12(1):2681. doi: 10.1038/s41467-021-23003-4 33976210 PMC8113569

[44] Wang J, Lu W, Zhang J, Du Y, Fang M, Zhang A, et al. Loss of TRIM29 mitigates viral myocarditis by attenuating PERK-driven ER stress response in male mice. Nat Commun. 2024;15(1):3481. doi: 10.1038/s41467-024-44745-x 38664417 PMC11045800

