# Supplementary figures and images for: Circovirus Rep evades immune restriction by disrupting cGAS oligomerization and phase separation
Source: PLoS Pathog. 2025 Jun 16;21(6):e1013244. doi: 10.1371/journal.ppat.1013244 (PMC12201654; doi:10.1371/journal.ppat.1013244)

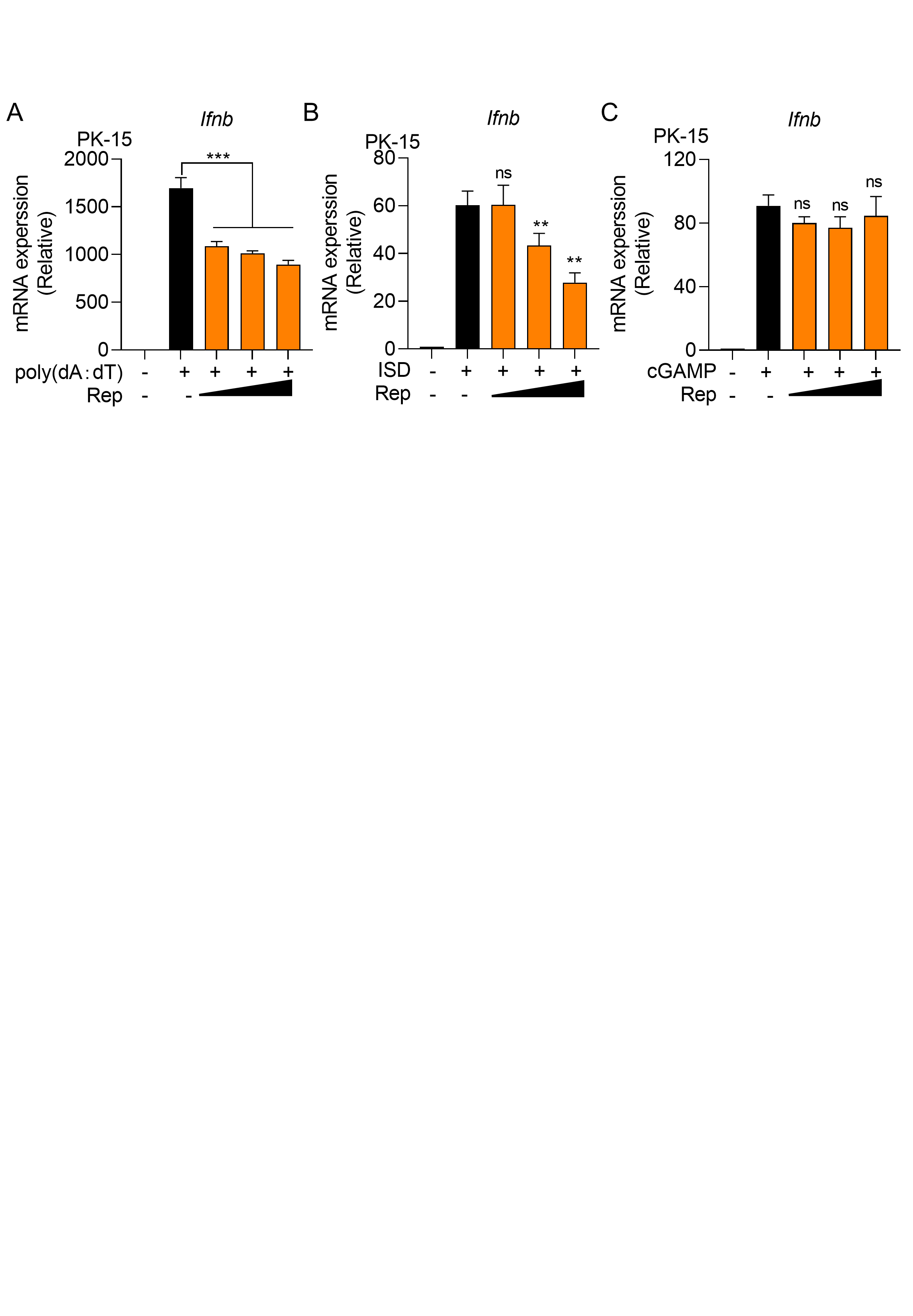

Supplement: S1 Fig — (A to C) PK-15 cells were transfected with increasing doses of PCV2d Rep for 24 h. Cells were stimulated with poly (dA:dT) (1 μg/mL) (A), ISD (2 μg/mL) (B) and 2’3’-cGAMP (1 μg/mL) (C). After another 12 h, cells were harvested for for RNA extraction and RT-PCR analysis of Ifnb expression. Data are represented as means ± SD from three biological replicates. ns, no significance, ***p < 0.001, Student’s t-test. (TIF) [file ppat.1013244.s001.TIF]

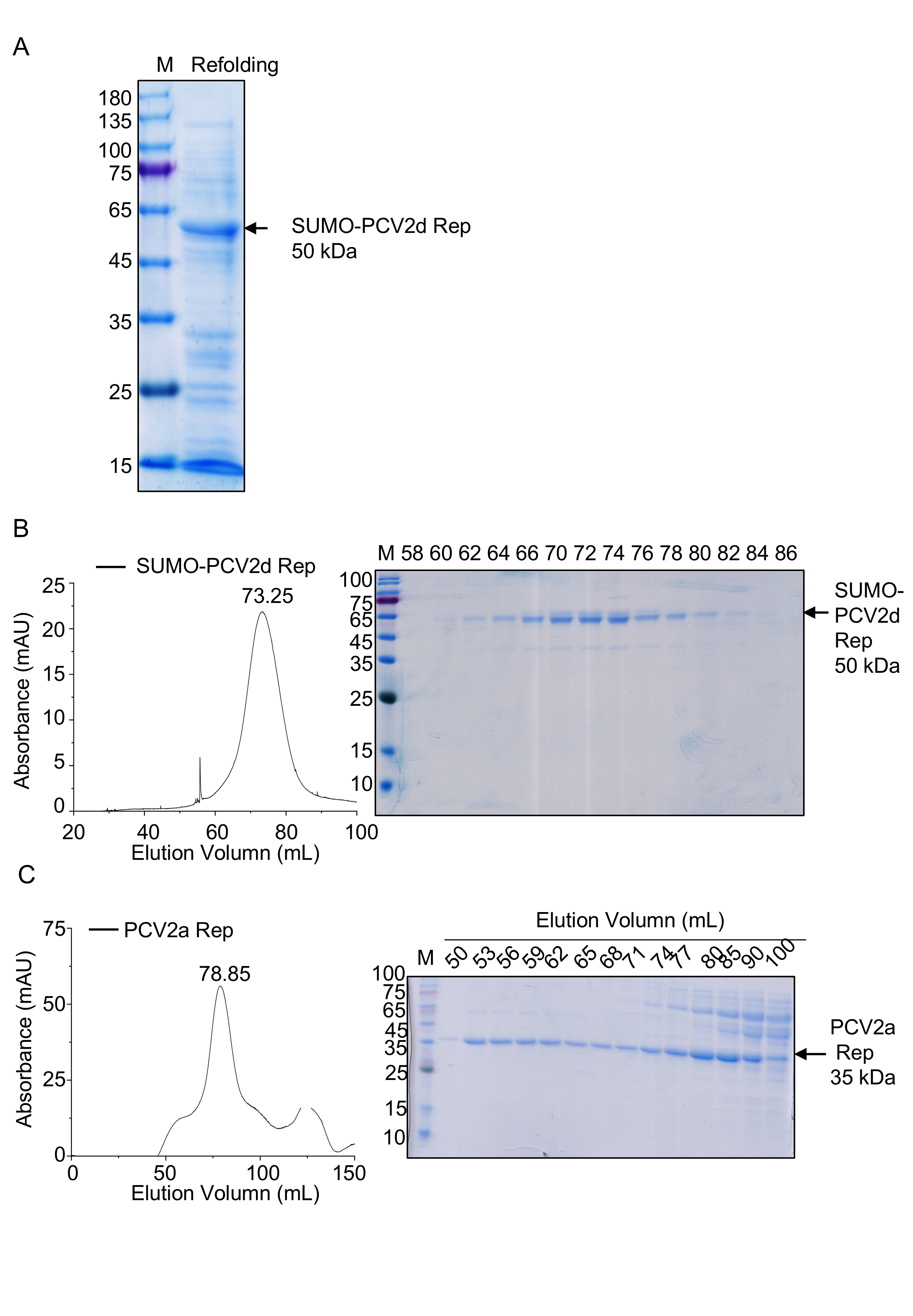

Supplement: S2 Fig — (A) Purification of refolding of full-length PCV2d Rep protein in vitro. (B) The detection of refolding of SUMO-tagged PCV2d Rep protein by gel-filtration chromatography. (C). Purification of PCV2a Rep protein by gel-filtration chromatography. (TIF) [file ppat.1013244.s002.TIF]

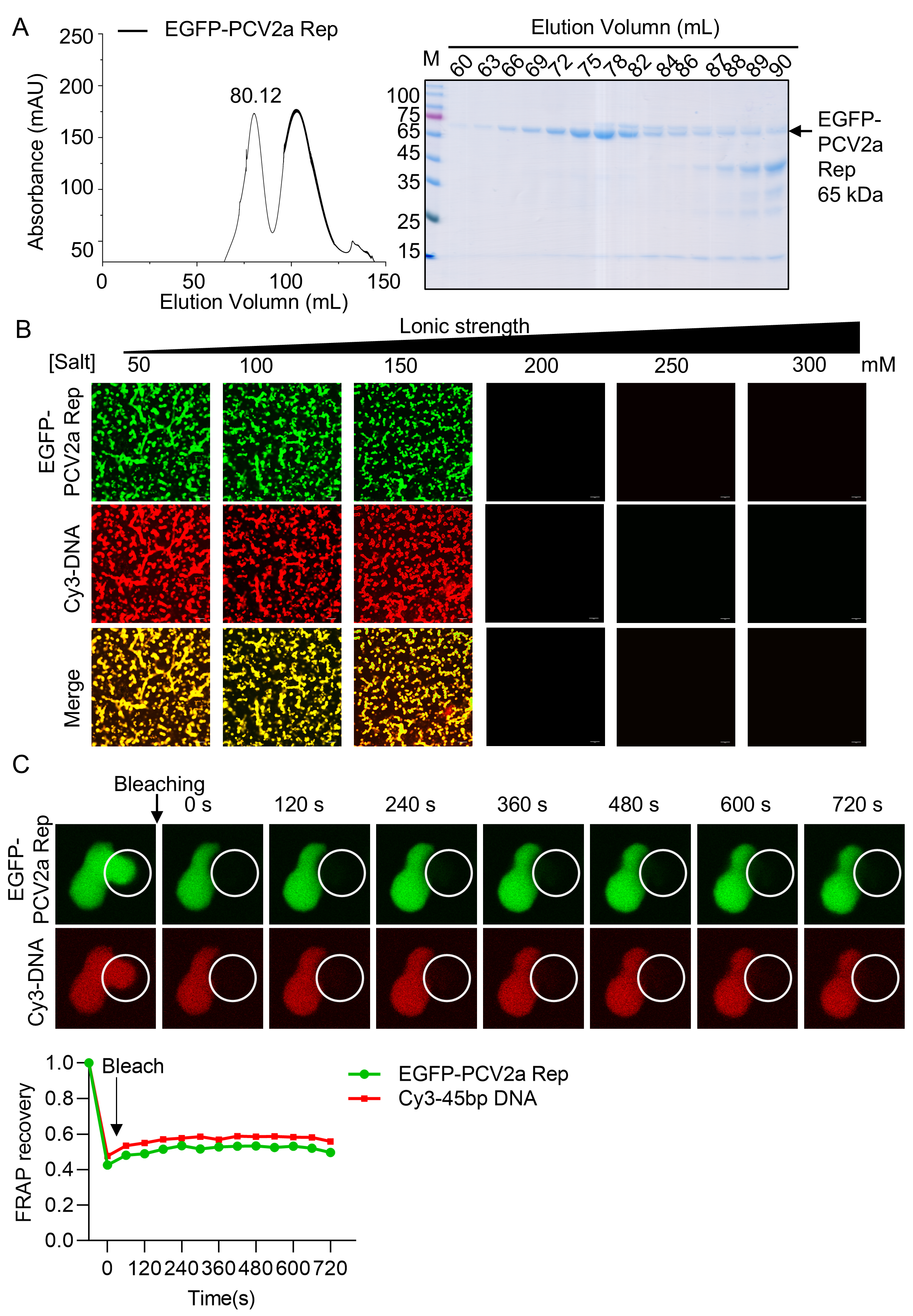

Supplement: S3 Fig — (A) Purification of EGFP-tagged PCV2a Rep protein by gel-filtration chromatography. (B) Phase separation of EGFP-PCV2a Rep with Cy3–45 bp DNA. (C) Fluorescence recovery of the PCV2a Rep-DNA condensate after photobleaching (FRAP). Bleaching was performed at the indicated time points after PCV2a Rep (10 μM) and DNA (5 μM) were mixed and the recovery was occured at 25 °C. Scale bar, 5 μm. The maximal fluorescence intensity was normalized to 1. (TIF) [file ppat.1013244.s003.TIF]

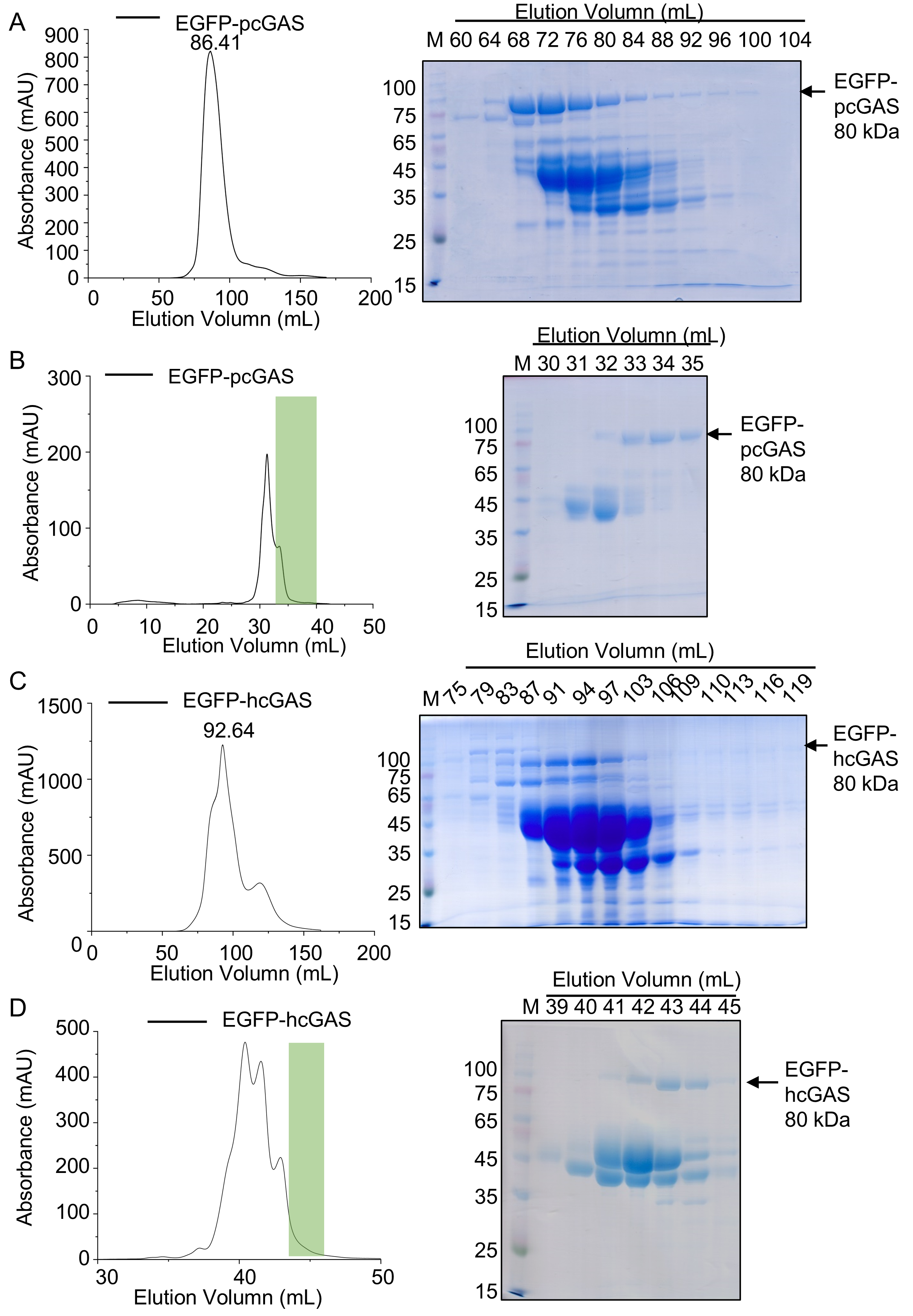

Supplement: S4 Fig — (A and B) Purification of EGFP-tagged full-length pcGAS protein by gel-filtration chromatography (A) and ion exchange (B). (C and D) Purification of EGFP-tagged full-length hcGAS protein by gel-filtration chromatography (C) and ion exchange (D). (TIF) [file ppat.1013244.s004.TIF]

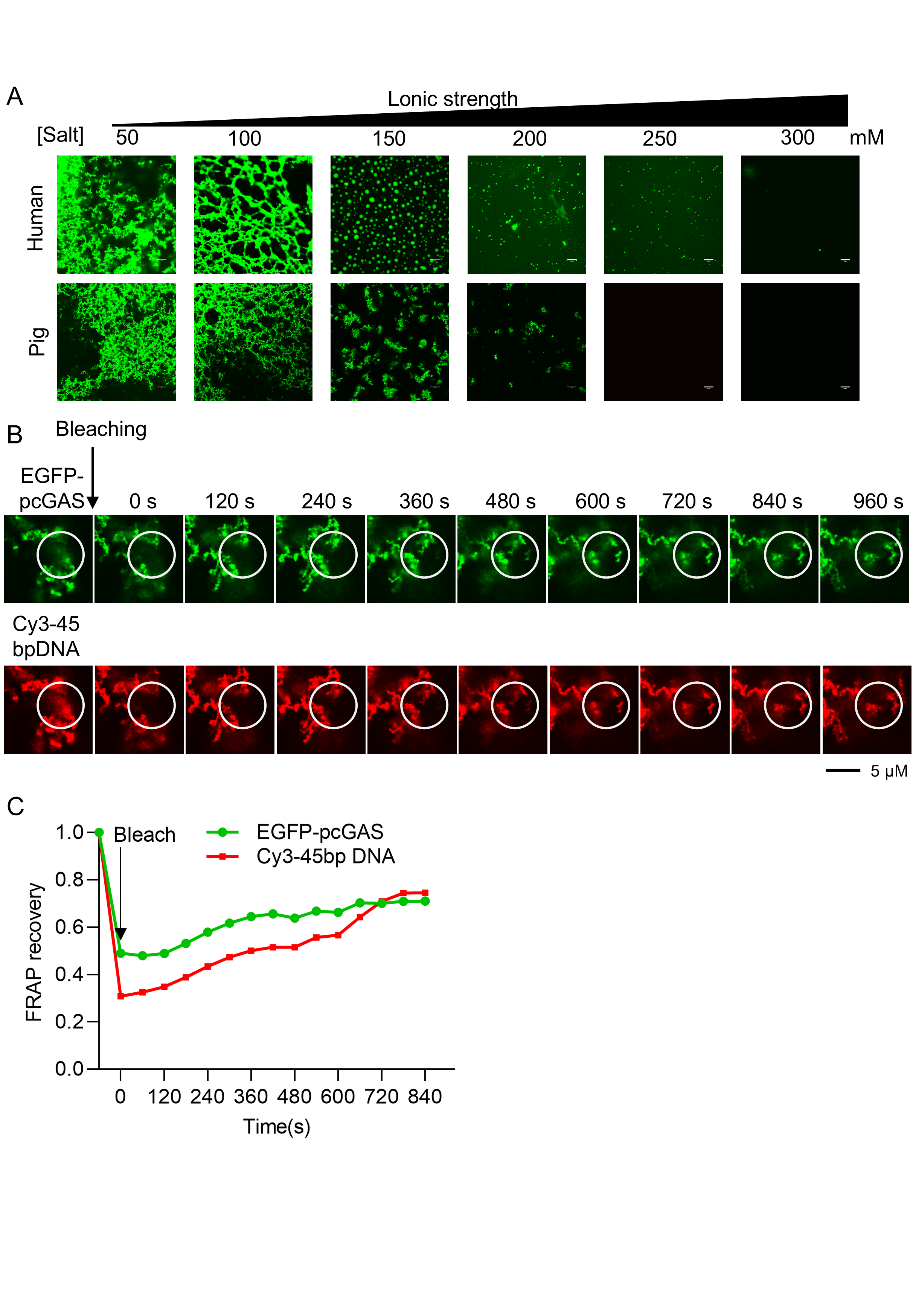

Supplement: S5 Fig — (A) Phase separation was induced with recombinant hcGAS (10 μM) and pcGAS (10 μM) and 45 bp dsDNA (5 μM) in buffer with varying salt concentration. Scale bar, 10 μm. (B and C) Fluorescence recovery after photobleaching (FRAP) of pcGAS–DNA phase-separated condensates. Bleaching was performed at the indicated time points after pcGAS (10 μM) and DNA (5 μM) were mixed and the recovery was allowed to occur at 25 °C. Scale bar, 5 μm. The maximal fluorescence intensity was normalized to 1. The statistical values are shown in C. (TIF) [file ppat.1013244.s005.TIF]

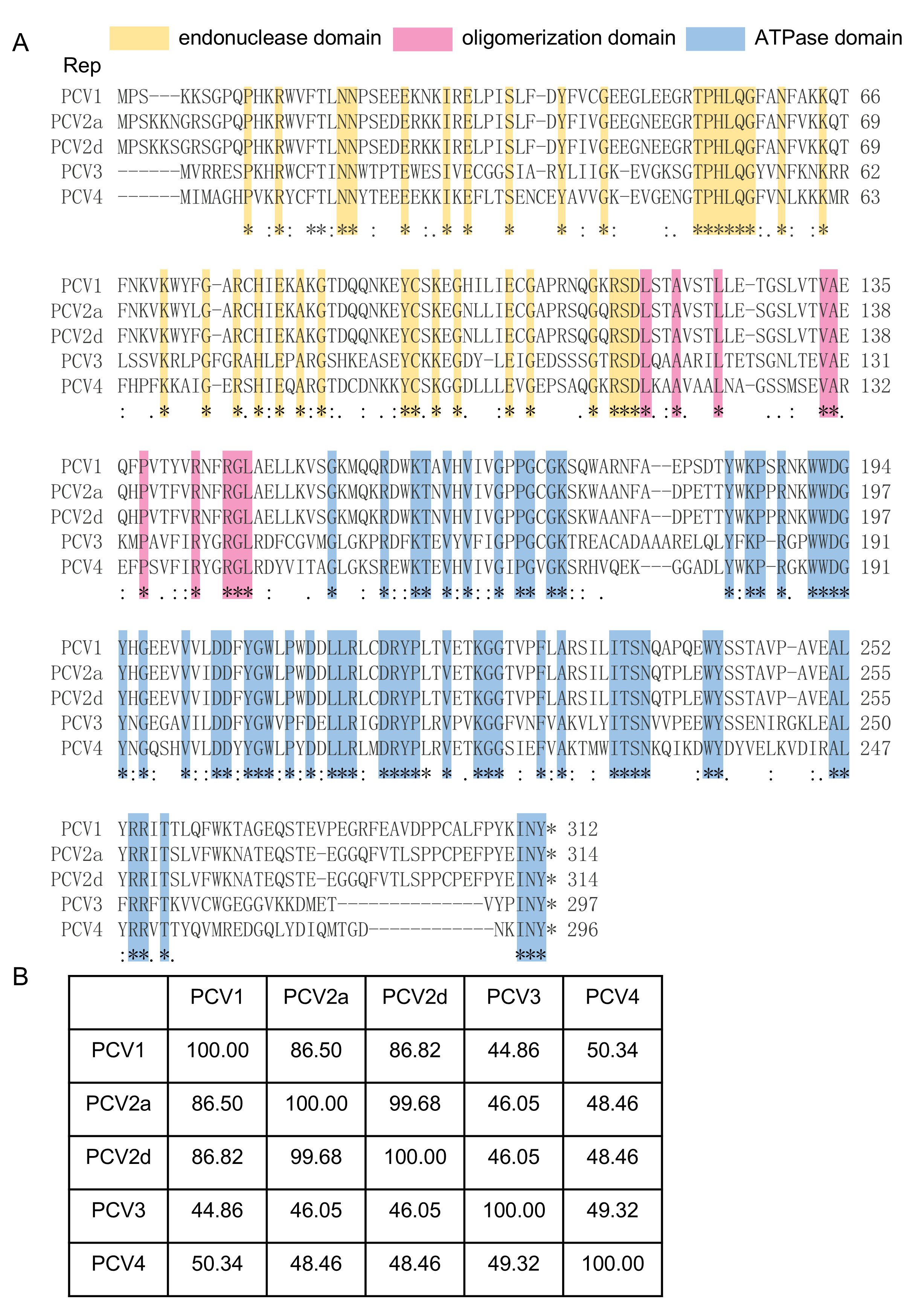

Supplement: S6 Fig — (A and B) PCV1 Rep (Genbank: KC447455.1), PCV2a Rep (Genbank: FJ870968.1), PCV2d Rep (Genbank: ON500676.1), PCV3 Rep (Genbank: MN788125.1) and PCV4 Rep (Genbank: MT311854.1) were aligned by Clustal Omega algorithm. Schematic of Rep with the endonuclease domain (pastel yellow), oligomerization domain (pastel red), and ATPase domain (pastel blue) labeled. (B). Comparison of amino acids identity (%) among different PCV Rep proteins. (TIF) [file ppat.1013244.s006.tif]

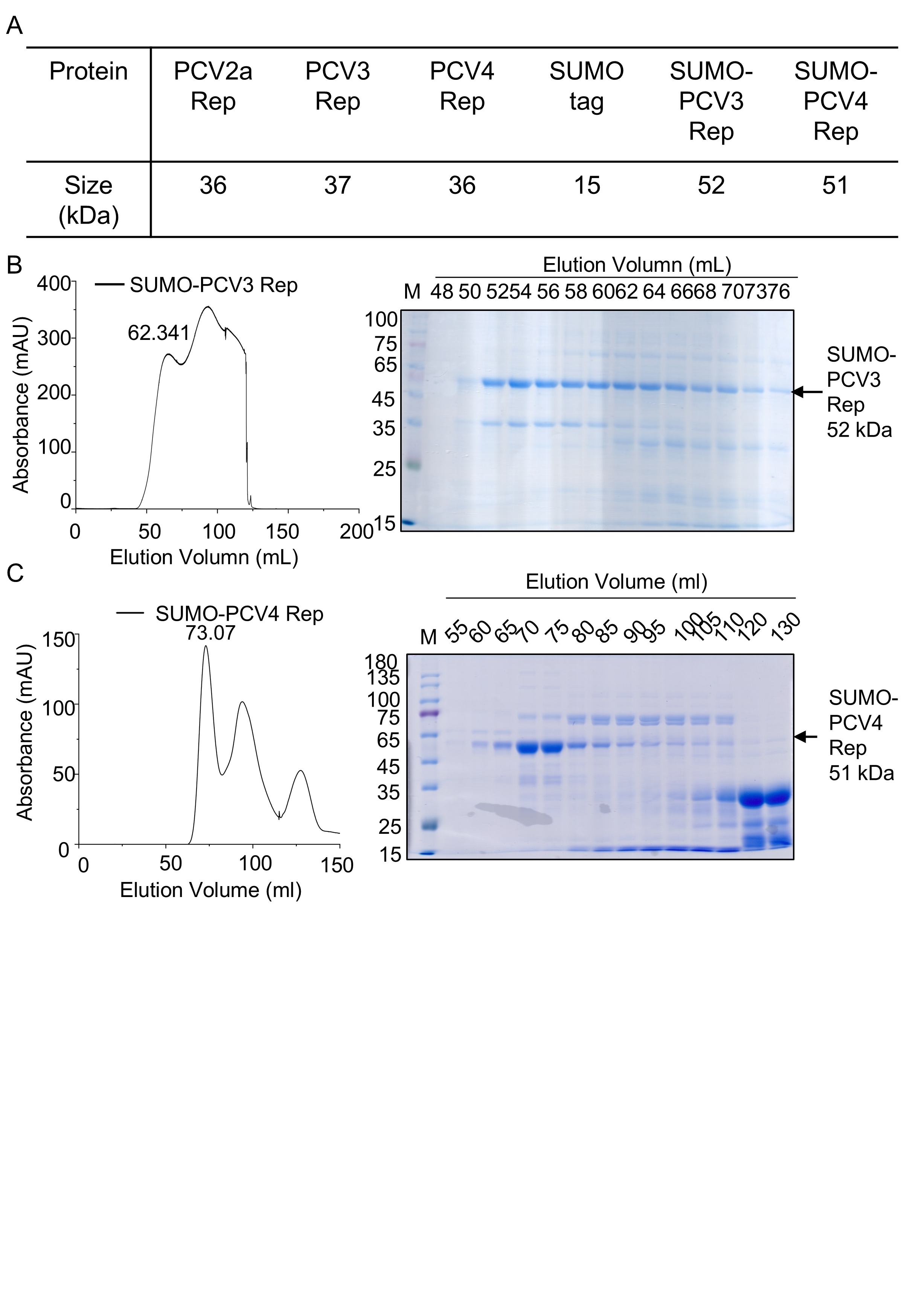

Supplement: S7 Fig — (A) The table of molecular weights of PCV2a, SUMO-PCV3 Rep, and SUMO-PCV4 Rep recombinant proteins using NovoPro (https://novopro.cn/tools/protein-sds-page-mw.html). (B and C) Purification of SUMO-PCV3 Rep (B) and SUMO-PCV4 Rep (C) protein by gel-filtration chromatography. (TIF) [file ppat.1013244.s007.TIF]

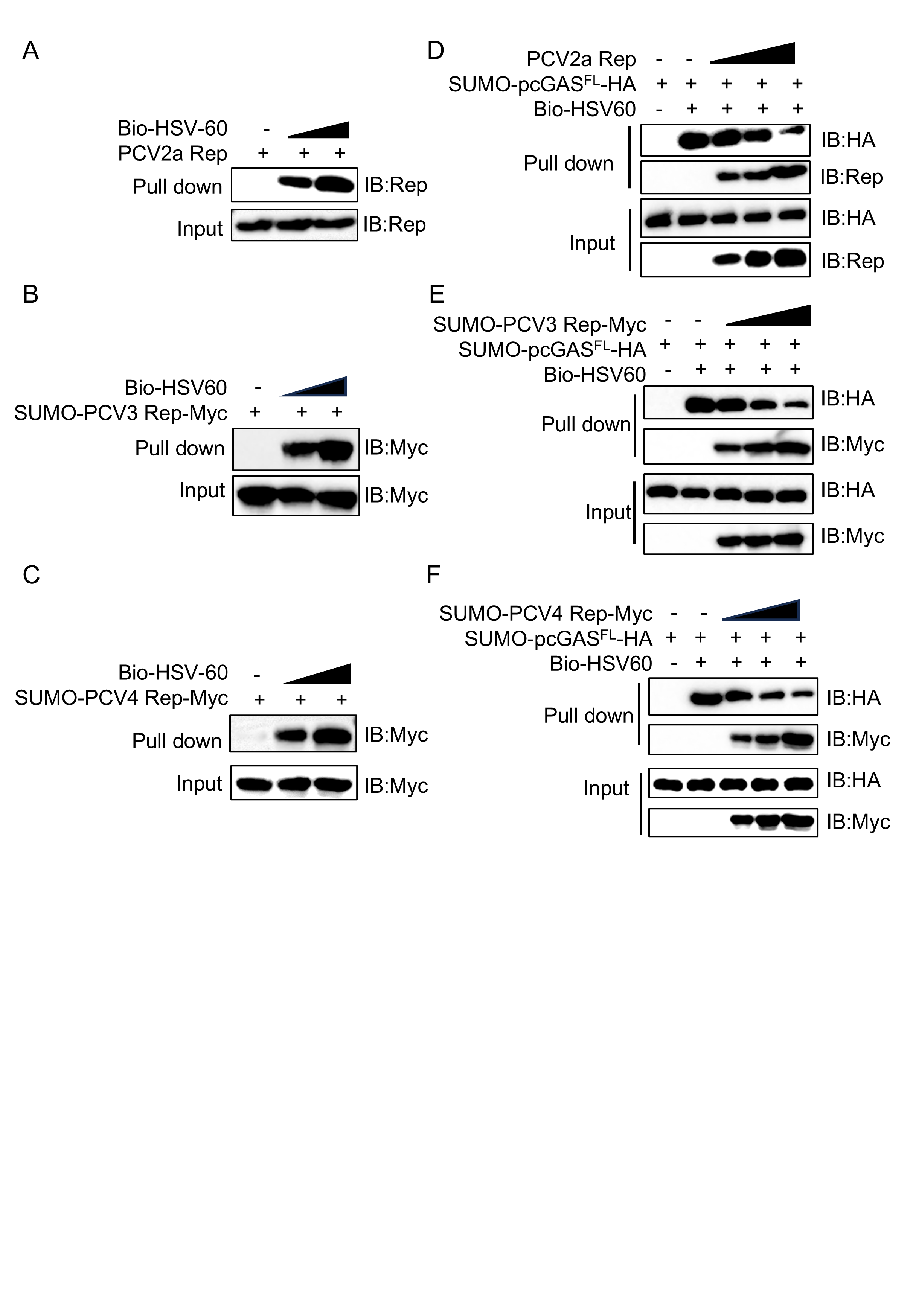

Supplement: S8 Fig — (A-C) Pull-down assay for assessment of the interactions between PCV2a (A), PCV3 (B) and PCV4 (C) Rep protein and biotin-HSV60 (40 μg/mL) DNA. After mixing the purified recombinant proteins in NP-40 buffer at 4°C for 6 h, the mixed buffer was immunoprecipitated with Streptavidin mAb. The immunoprecipitated complex was analyzed by immunoblotting with the indicated antibodies. (D-F) Pull-down assay for assessment of the interactions between PCV2a Rep (C), SUMO-PCV3 Rep-Myc (E) or SUMO-PCV4 Rep-Myc (F) or pcGAS full length protein and biotin-HSV60 (40 μg/mL) DNA. (TIF) [file ppat.1013244.s008.TIF]

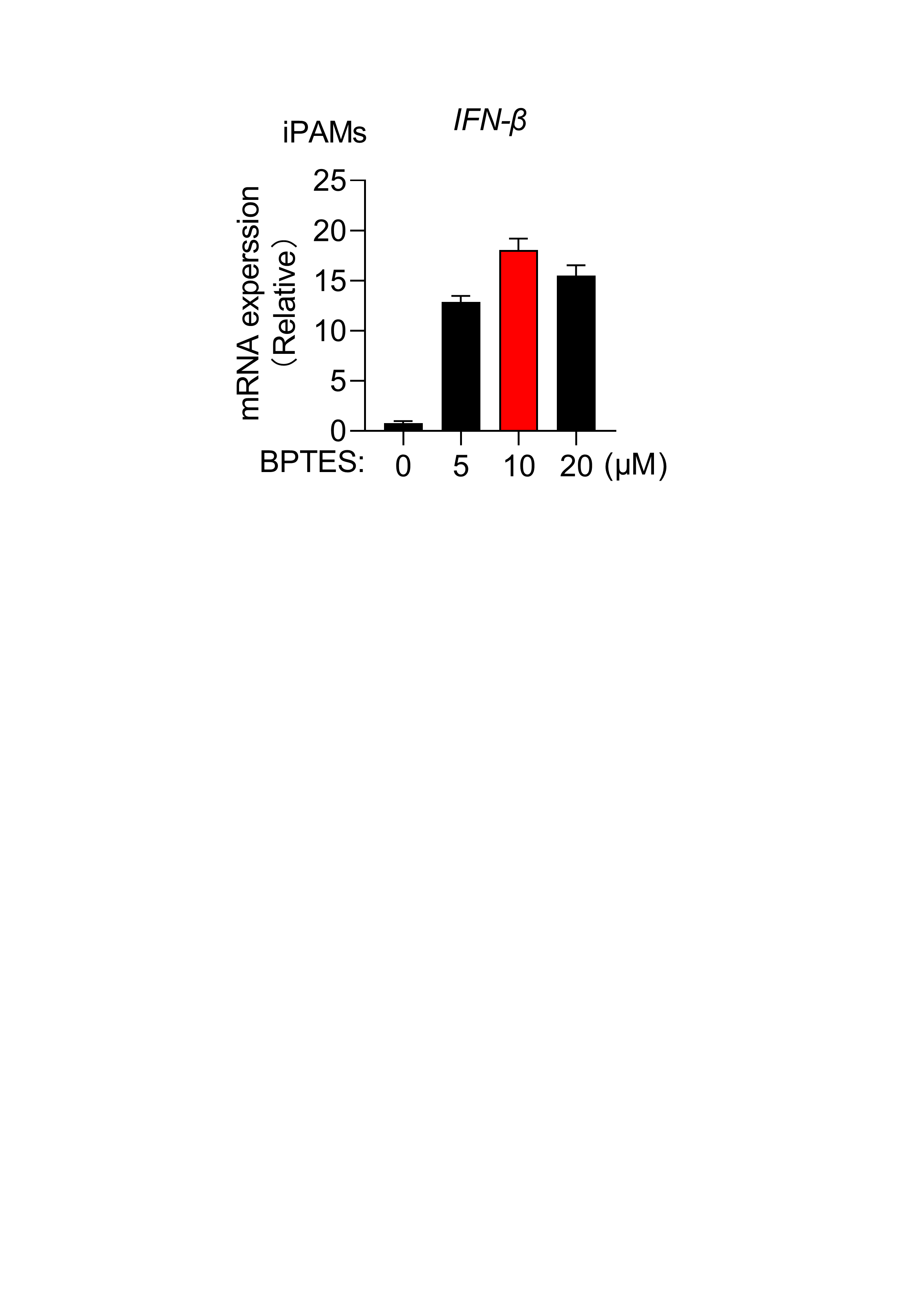

Supplement: S9 Fig — (TIF) [file ppat.1013244.s009.TIF]

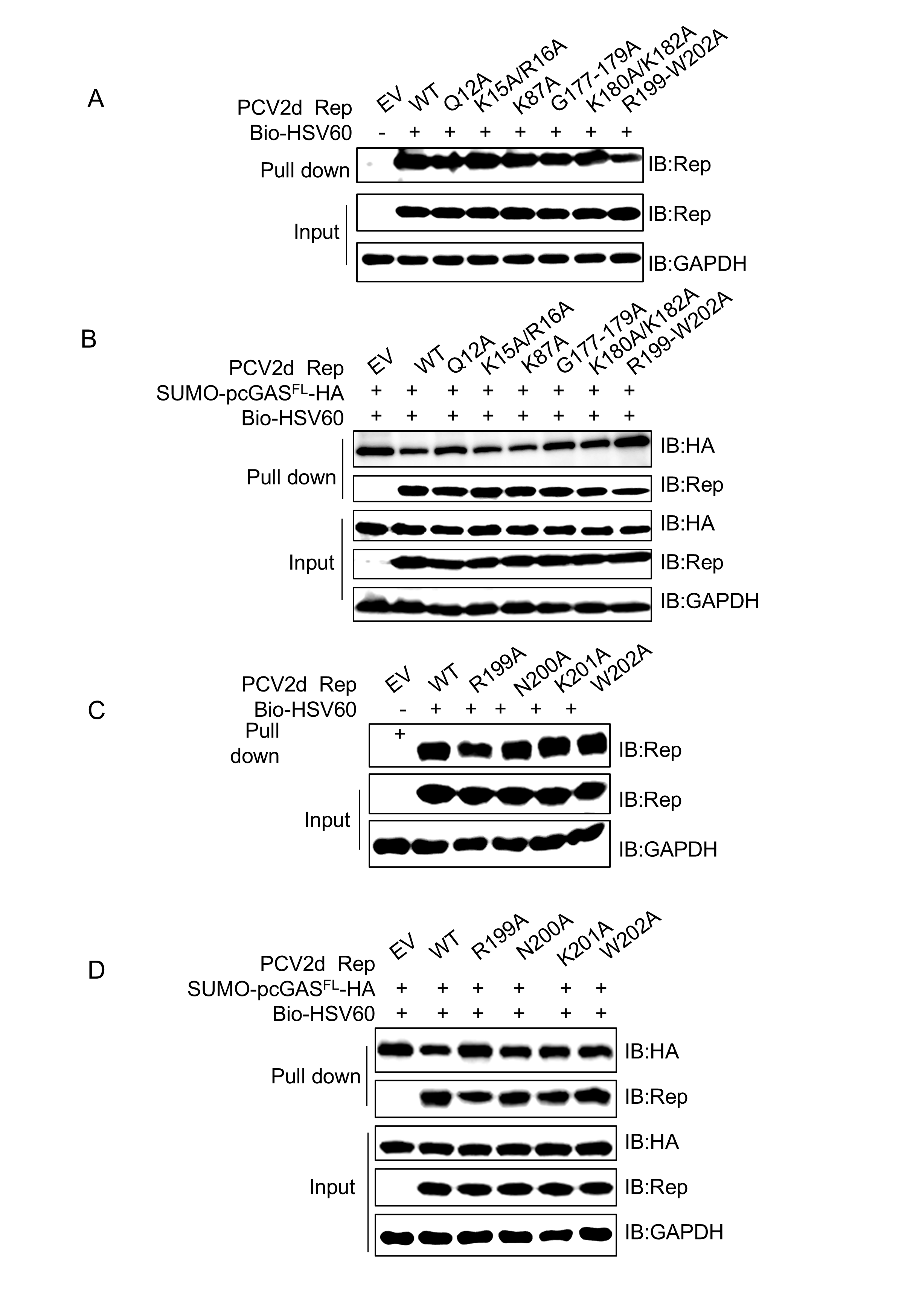

Supplement: S10 Fig — (A) Pull-down assay for assessment of the interactions between the whole cell lysate in HEK-293T cells transfected with a plasmid encoding different mutations (wild-type, Q12A, K15A/R16A, K87A, G177-179A, K180A/K182A or R199-W202A) of PCV2d Rep and biotin-HSV60 (40 μg/mL) DNA. (B) Pull-down assay for assessment of the interactions between the whole cell lysate in HEK-293T cells transfected with a plasmid encoding different mutations (wild-type, Q12A, K15A/R16A, K87A, G177-179A, K180A/K182A or R199-W202A) of PCV2d Rep or full-length pcGAS protein and biotin-HSV60 (40 μg/mL) DNA. (C) Pull-down assay for assessment of the interactions between the whole cell lysate in HEK-293T cells transfected with a plasmid encoding different mutations (wild-type, R199A, N200A, K201A or W202A) of PCV2d Rep and biotin-HSV60 (40 μg/mL) DNA. (D) Pull-down assay for assessment of the interactions between the whole cell lysate in HEK-293T cells transfected with a plasmid encoding different mutations (wild-type, R199A, N200A, K201A or W202A) of PCV2d Rep or full-length pcGAS protein and biotin-HSV60 (40 μg/mL) DNA. (TIF) [file ppat.1013244.s010.TIF]

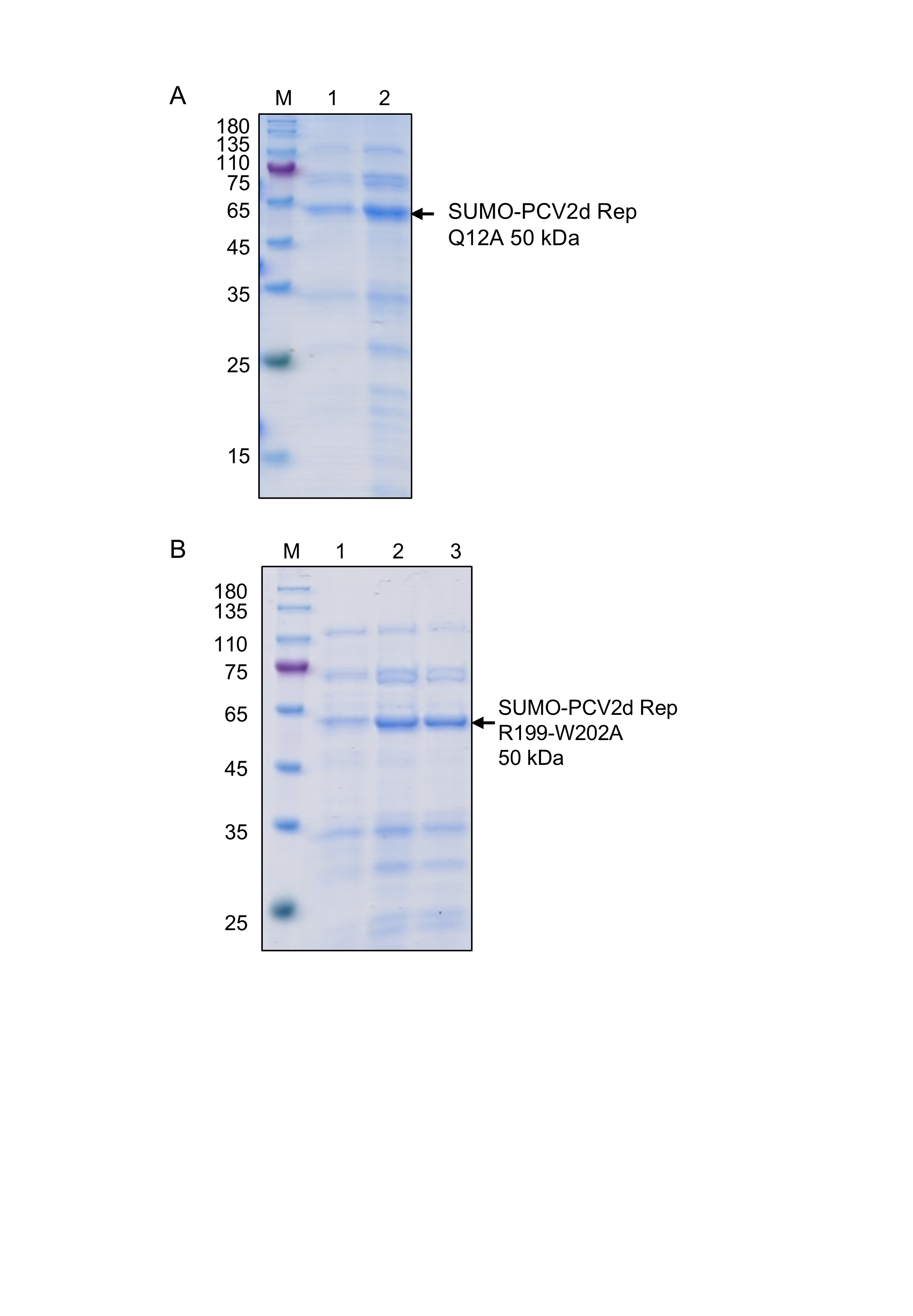

Supplement: S11 Fig — (A and B) Purification of recombinant Q12A (A) and R199-W202A (B) mutation PCV2d Rep proteins by Ni-NTA. (TIF) [file ppat.1013244.s011.TIF]
